# Upgrading biocrude oil into sustainable aviation fuel using zeolite-supported iron-molybdenum carbide nanocatalysts

**DOI:** 10.1126/sciadv.adu5777

**Published:** 2025-06-27

**Authors:** Siying Yu, Haozhen He, Sabrina Summers, Zhibin Yang, Buchun Si, Runnan Gao, Anran Song, Joshua Heyne, Yuanhui Zhang, Hong Yang

**Affiliations:** ^1^ Department of Chemical and Biomolecular Engineering, University of Illinois Urbana-Champaign, 600 South Mathews Ave., Urbana, IL 61801, USA.; ^2^ Department of Agricultural and Biological Engineering, University of Illinois Urbana-Champaign, Urbana, IL 61801, USA.; ^3^ Bioproducts, Science, and Engineering Laboratory, Washington State University, Richland, WA, USA.; ^4^ Laboratory of Environment-Enhancing Energy (E2E), Key Laboratory of Agricultural Engineering in Structure and Environment, Ministry of Agriculture, College of Water Resources and Civil Engineering, China Agricultural University, Beijing, China.; ^5^ DOE Center for Advanced Bioenergy and Bioproducts Innovation, University of Illinois Urbana-Champaign, Urbana, IL 61801, USA.; ^6^ Energy Processes and Materials Division, Energy and Environment Directorate, Pacific Northwest National Laboratory, Richland, WA 99352, USA.; ^7^ Materials Research Laboratory, The Grainger College of Engineering, University of Illinois Urbana-Champaign, Urbana, IL 61801, USA.; ^8^ Department of Chemistry, University of Illinois Urbana-Champaign, 600 South Mathews Ave., Urbana, IL 61801, USA.

## Abstract

Food waste is an underdeveloped source for production of sustainable aviation fuel (SAF). Now, there is no certified conversion process of food waste for SAF by American Society for Testing and Materials (ASTM). We report the use of zeolite-supported molybdenum carbide nanocatalysts in upgrading biocrudes, produced from food wastes through HTL, into SAF precursors. Our data show a complete removal of oxygen from the biocrude through hydrodeoxygenation and a higher heating value of 46.5 MJ/kg, which is comparable to that of Jet A (46.1 MJ/kg). The prescreening tests (tier alpha and beta) show the average carbon number of the distillation cut (150° to 230°C) of upgraded fuel is 10.6, close to the value of 11.4 for average conventional jet fuel, and the specifications of properties including surface tension, viscosity, heating value, flash point, and freezing point were found to meet the standards of SAF. The metal carbide nanocatalysts were reusable in upgrading tests, and the activity of deoxygenation was retained.

## Teaser

Biocrude converted from food waste via HTL was upgraded to sustainable aviation fuel using iron-molybdenum carbide-ZSM-5 catalyst.

## INTRODUCTION

Large-scale production of sustainable aviation fuel (SAF) (*1*, *2*) from wet wastes is crucial to achieve the target of reaching net zero carbon emission by 2050 (*3*). This is because SAF has less safety concern than other alternative energy carriers such as hydrogen and has a volumetric energy density (34 MJ/liter) 4.0 to 6.9 times higher than hydrogen. Thus, SAF has been considered the only net zero mechanism for long-haul wide-body aircrafts over the next several decades (*4*). In addition, SAFs are drop-in compatible with all existing infrastructure and aircraft. Therefore, the existing extensive infrastructure in aviation and their current regulatory compliance can be leveraged under the aviation decarbonization strategy with SAFs.

Triglyceride, lignocellulosic biomass, and sugar/starch are three typical sources for SAF production (*5*). While lignocellulosic biomass as feedstock is cheaper than the other two, it requires expensive and complicated processing (*6*, *7*). Sugar including starch is mainly used for generating light fuels consisting of short carbon chain components and requires multistep conversions, including fermentation (to produce alcohols), dehydration, oligomerization, and hydrogenation (*8*). Catalytic hydrotreating of triglyceride feedstock, on the other hand, can be a relatively simple approach, in which hydrodeoxygenation (HDO), hydroisomerization, and hydrocracking can simultaneously take place (*9*). Other than the benefit of facile processing, using triglyceride-based wet waste as feedstock also increases energy recovery (*10*, *11*), valorizes unwanted bioresources, and alleviates water contamination caused by organic substances (*12*).

A major class of biocrude oil is made from triglyceride-based wet wastes through hydrothermal liquefaction (HTL) (*9*, *13*–*15*) and will be used in this study for upgrading to SAFs. A standard set of specifications derived from conventional fuels has been established to prescreen if an upgraded product is suitable as SAF precursor (*16*). An important criterion is, unlike diesel fuel, a SAF precursor should not contain oxygen, which must be removed to prevent corrosion of jet engine by oxygen-containing compounds such as organic acids. Oxygen also adversely increases density and viscosity of a biocrude oil. The optimal number of carbons in hydrocarbons for aviation fuel, which affects the flash and freezing point of a fuel, is typically in the range of C8 to C16. The preferred jet fuel consists of oxygen-free organics of paraffins, isoparaffins, naphthenes, and aromatics (*17*). Higher heating value (HHV), which can be estimated by elemental (e.g., carbon, nitrogen, oxygen, and hydrogen) contents of a fuel, is another key parameter and should be close to that of Jet A. In short, it is essential to develop scalable and highly reactive catalysts and to demonstrate their use in the production of SAF precursors using real biocrude based on the studies using model compounds (*18*, *19*).

Transition metal carbides (TMCs), such as beta phase molybdenum carbide (β-Mo_2_C), have unique catalytic properties because of their platinum-like electronic structures (*20*, *21*). Recently, β-Mo_2_C catalysts were shown to exhibit HDO activity comparable to platinum and palladium using model compounds such as oleic acid and guaiacol (*18*, *22*–*24*). Both metal-like hydrogen adsorption sites and Brønsted acid hydroxyl sites on β-Mo_2_C surface were thought to be beneficial to the HDO reaction (*24*, *25*). These carbide catalysts also resist poisoning and corrosion from impurities under harsh conditions using model compounds (*22*–*24*, *26*, *27*). While these studies are extremely useful, model reactions are far from sufficient to evaluate the suitability of wet waste–derived biocrude for SAF because the complexity in composition of biocrude oil cannot be replicated by model compounds (*22*, *23*). In addition, using model compounds one cannot evaluate the whole ranges of specifications of the fuel, including viscosity, freezing point, flash points, to name a few (*28*). Hydrodeoxygenation of HTL biocrude oil from food waste has been demonstrated using alumina-supported metal alloy catalysts, including CoMo and NiMo. Sulfided CoMo/Al_2_O_3_ and NiMo/Al_2_O_3_ catalysts were used previously to produce SAF from HTL biocrudes of food waste at 400°C, 1500 psi, and 0.5-hour^−1^ weight hour space velocity (*13*). The upgraded biocrude achieved 93% deoxygenation using CoMo catalysts and 91% using NiMo catalysts, increasing to 98% after taking the jet fuel cut. In addition, a two-stage hydrotreating approach using CoMo for demetallization and mild hydrotreating, followed by NiMo for more severe hydrotreating, achieved 97% deoxygenation of HTL biocrude from food waste, producing a diesel-rich product (*9*). Thus, investigation of Mo_2_C-based nanocatalysts in the upgrading of real biocrude oil is much needed to establish the proper processing pathways to the SAF production from wet waste–derived biocrudes (*29*, *30*).

Herewith we report the use of zeolite-supported iron–β-Mo_2_C nanocatalysts in the upgrading of real biocrude oil, derived from wet food waste by HTL, to a SAF precursor that meets all essential criteria in the FAA standard tier beta test. In our approach, β-Mo_2_C nanocatalysts were dispersed onto zeolite supports (*25*, *31*, *32*) to enhance the catalyst dispersion, thus giving rise to a high HDO activity (*19*). Zeolite Socony Mobil–5 (ZSM-5) was chosen as the catalyst support for its capability to dispersing molybdenum carbide (*25*) and to produce high aromatic yields with low coke formation (*33*). Iron (Fe) was used as the cocatalyst and incorporated into the metal carbide to promote cracking (*34*–*38*). Previously, Fe/ZSM-5 catalysts were reported to exhibit good cracking performance and a Fe loading amount as low as 5 weight % (wt %) could be sufficient to modify the overall acidity (*37*). The 3-nm Fe-containing β-Mo_2_C nanoparticle catalyst on ZSM-5 (Fe-Mo_2_C/ZSM-5) was synthesized via carburization method at 600°C. This catalyst exhibited excellent HDO activity, resulting in complete removal of oxygen from the biofuel and an increase in hydrogen-to-carbon atomic (H/C) ratio to 1.96. Tier beta test indicates upgraded biocrude oil satisfied all key specifications of SAF prescreening standards, including surface tension (at 22°C), density (at 15°C), viscosity (at −20° and −40°C), lower heating value (LHV), flash point, and freezing point. In this context, we successfully produced 100% SAF precursors from food waste–derived biocrude oils using non-noble metal nanocatalysts.

## RESULTS

### Compositional and structural characterization of Fe-Mo_2_C/ZSM-5

**Fig. 1.**
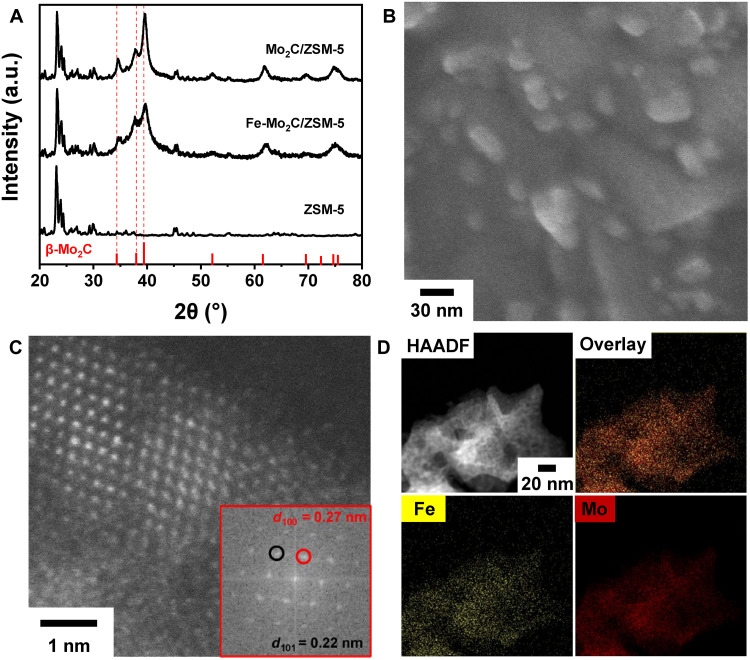
Characterizing the structures of Fe-Mo_2_C/ZSM-5 catalyst. (**A**) XRD patterns, (**B**) SEM, (**C**) HAADF-STEM, and (**D**) STEM-EDS elemental mapping of Fe and Mo. The XRD patterns of Fe-free Mo_2_C/ZSM-5 and the commercial ZSM-5 are included for comparison. The pattern of β-Mo_2_C is drawn using the database value (PDF#35-0787). Inset in (C) is the fast Fourier transform image of the corresponding area.

The Fe-Mo_2_C/ZSM-5 catalyst was synthesized at 600°C via the carburization method. For safety consideration, freshly made Mo_2_C catalyst was passivated using 2% O_2_ in the tube furnace before the characterizations and subsequent catalytic studies (see Materials and Methods for detail). The atomic ratio of Fe to Mo in the precursor is 5%. The stated amount of Fe cocatalyst was chosen on the basis of the prior study, in which 5 wt % on ZSM-5 provided the suited acidity for cracking with low coking effect (*25*, *33*–*38*). Our experience study indicates a temperature of 600°C or above was required to produce Mo_2_C. Inductively coupled plasma optical atomic emission spectroscopy (ICP-OES) shows the catalyst contains 1.30 wt % Fe and 41.9 wt % Mo, which is close to the nominal amount of 1.26 wt % Fe and 43.1 wt % Mo in the reactant mixture (table S1). X-ray diffraction (XRD) study indicates the as-made catalyst consists of phase-pure β-Mo_2_C on ZSM-5, with no diffraction from Fe-containing species (Fig. 1A). Mo_2_C nanocatalyst without Fe was made following the same synthetic procedure except without the addition of iron (III) nitrate. This Fe-free sample exhibits enhanced crystallinity of Mo_2_C, judging by the full width at half maximum (FWHM) and peak intensity (Fig. 1A). The addition of Fe effectively reduced the crystal domain size of Mo_2_C. The FWHM and positions of XRD peaks for ZSM-5 are similar in the three samples, suggesting no major change in zeolite structure. This structural stability is beneficial for the dispersion of metal precursors and the formation of metal carbide nanoparticle catalysts (*25*, *39*). Scanning electron microscopic (SEM) study shows the ZSM-5 particles have an average size of ~2 μm, which are made of aggregates of ~200-nm crystallites (fig. S1). This hierarchical morphology retained for Fe-Mo_2_C/ZSM-5 catalysts (fig. S2).

The Mo_2_C nanoparticle catalysts were further examined by both SEM (Fig. 1B) and high-angle annular dark field scanning transmission electron microscopy (HAADF-STEM) (Fig. 1C). The microscopic data show that the size of catalyst particles can be as large as ~30 nm, although the primary nanoparticles are only a few nanometers in size. The XRD Rietveld refinement data indicate the crystal domain size is about 3.0 nm (fig. S3 and table S2). The small sub–5-nm Mo_2_C crystallites were observed to disperse evenly over the ZSM-5 support, judging by the transmission electron microscopy (TEM) and STEM micrographs (figs. S4 and S5). The fast Fourier transform analysis indicates the lattice observed in HAADF-STEM has a *d*-spacing of 0.27 nm, which correspond to Mo_2_C (100) and of 0.22 nm to the (101) facet (Fig. 1C and fig. S6). These results show the carburization could successfully produce carbide nanoparticles with size optimal for catalysis, which is advantageous over the commercial Mo_2_C (figs. S7 and S8). STEM energy dispersive x-ray spectroscopy (EDS) shows both Fe and Mo elements are dispersed uniformly across the catalyst particles. (Fig. 1D and fig. S9).

### Upgrading and characterization of biocrude oil

**Fig. 2.**
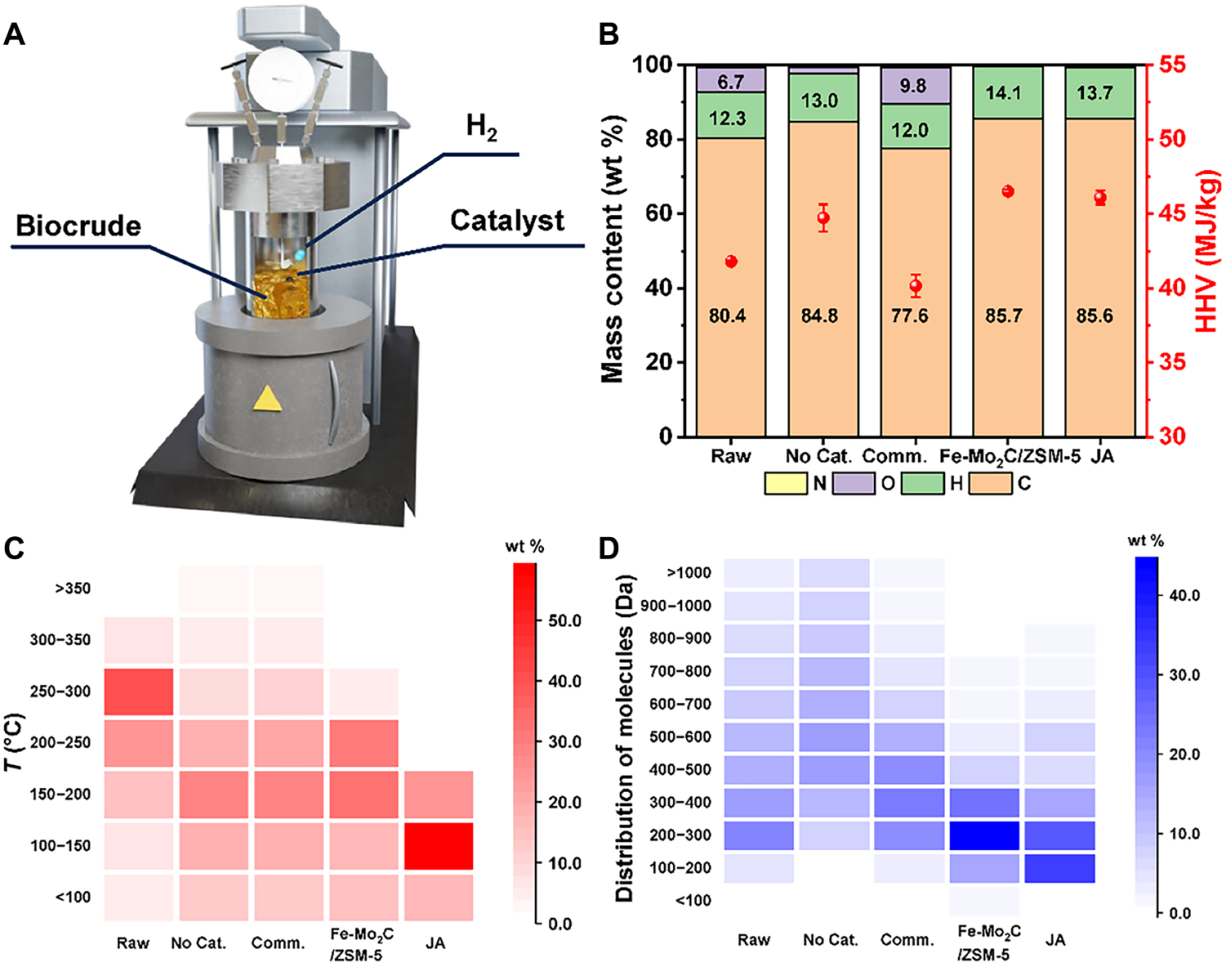
Upgrading and characterization of the biocrude oil and its upgraded products. (**A**) Illustration of the experimental setup. (**B**) Elemental analysis, (**C**) boiling point distribution based on TGA analysis, and (**D**) number-weighed molecular weight (Da) distribution for upgraded oils using Fe-Mo_2_C/ZSM-5. Data of untreated biocrude oil (Raw), upgraded oils without catalyst (No Cat.) and with commercial Mo_2_C (Comm.), and Jet A (JA) are included for comparison.

The upgrading of biocrude oil was performed using a Parr reactor as illustrated in Fig. 2A. The biocrude oil derived from the HTL process was mixed with freshly prepared Fe-Mo_2_C/ZSM-5 catalyst at a catalyst/biocrude mass ratio of 6:100. All catalytic upgrading was conducted at 400°C in pressurized H_2_ for 2 hours, unless otherwise stated. Elemental analysis data indicate the oxygen content in the oil decreased from 6.74 to ~0 wt % after the upgrading using Fe-Mo_2_C/ZSM-5, showing a complete deoxygenation (Fig. 2B and table S3). After the hydrodeoxygenation process, 6.6 ml (or 4.8 g) of SAF precursor could be obtained from 10 ml of the biocrude oil (density: 0.857 g/ml). This result corresponds to a conversion of about 56 wt % from pretreat biocrude to SAF precursor, similar to the conversion to hydrocarbon from volatile fatty acids derived from the biobased chemicals through the anaerobic digestion (*40*). The hydrogen content increased from 12.26 to 14.08 wt %, corresponding to an increase of HHV from 41.80 to 46.51 MJ/kg, which is comparable to that of Jet A (table S4). The substantial improvement in oil quality could also be readily observed from the color change of oils before and after the treatment (fig. S10). To understand the effects of processing variables such as temperature and hydrogen pressure, we conducted upgrading experiments in the absence of Fe-Mo_2_C/ZSM-5 catalyst while keeping all other conditions the same. The oxygen content decreased to 1.70 wt %, which was equal to about 75% of deoxygenation. The hydrogen content of oil, however, only slightly increased to 13.0 wt %. We analyzed the H/C ratio of the upgraded oil, which is a measure of the degree of saturation of organic compounds after the catalytic hydrogenation. Our results show after the treatment by Fe-Mo_2_C/ZSM-5 catalyst, the oil product exhibited an H/C ratio of 1.96, much higher than that of 1.82 if no catalyst was used. Noticeably, the upgraded oil exhibited the H/C ratio, thus the heating value, comparable to that of Jet A. The oil produced after treatment with commercial Mo_2_C, however, shows no improvement or even worse than the raw biocrude oil in H/C ratio and oxygen content, with 9.8 wt % O and 12.0 wt % H. Our study shows the nanometer-sized Mo_2_C particle and zeolite/Fe compositions in the Fe-Mo_2_C/ZSM-5 catalysts are necessary to achieve high HDO activity.

We further investigated the distribution of boiling point of the upgraded oils by performing thermogravimetric analysis (TGA) (fig. S11A). The weight percentage of upgraded oil after treatment using the Fe-Mo_2_C/ZSM-5 nanocatalysts are preferably in the low-temperature regions, with decreased distribution in temperature range above 250°C, as contrast to those treated without catalyst or with the commercial Mo_2_C (fig. S11B). The weight distribution heatmaps of different oils were obtained by calculating the integral of percentage weight over different temperature ranges (Fig. 2C and table S5). A decrease of weight distribution was observed in the range of 250° to 300°C for all oil samples after the catalytic treatment. This result suggests long carbon chain substances in the oils were cracked in pressurized H_2_ at 400°C. Weight distribution above 350°C slightly increases for upgraded oils using no catalyst or the commercial Mo_2_C, which could be caused by the polymerization reaction during upgrading. These two oil products also exhibit relatively uniform weight distributions across 100° to 300°C, while the upgraded oil using the Fe-Mo_2_C/ZSM-5 catalyst shows the predominant fraction is in the temperature range of 150° to 250°C. Because the wet waste–derived biocrude oil is composed of a mixture of different components, an overall weight distribution towards the lighter range increases the percentage of distillation cut that meets the SAF standards (table S6). In a different classification (fig. S11C), our catalytic treatment could upgrade the “diesel-like” raw biocrude oils into “kerosene/gasoline-like” fuels, noting the property of Jet A is regarded as gasoline-like (*41*).

Matrix-assisted laser desorption ionization time of flight mass spectrometry (MALDI-TOF-MS) was carried out to study the distribution of molecular weight of compounds in various oils (fig. S12 and table S7), which is directly related to the length of carbon chains. An interval of 100 Da was used in our calculation of the number-weighed molecular weight (Fig. 2D). The molecular weight distribution between 100 and 300 Da was given special attention, since carbon chains in the range of C8 to C16 are preferred for SAFs. In the raw biocrude oil, a total of 25.9 wt % was found to locate in this range of molecular weight distribution, while 8.0 and 22.9 wt % were found for the oil products after treatments without the catalyst and with commercial Mo_2_C, respectively. After the treatment with Fe-Mo_2_C/ZSM-5 catalyst, 59.4 wt % of the upgraded oil was found in this molecular weight distribution, which is comparable to that of Jet A (62.1 wt %). The upgraded oil has lower molecular weights in all intervals above 300 Da. Table S7 summarizes the number-averaged molecular weights (*M*_n_) and mass-averaged molecular weights (*M*_m_) of all oils tested. Upgraded oil using the Fe-Mo_2_C/ZSM-5 catalyst has a *M*_n_ of 254 Da, which is comparable with that of Jet A (242 Da) and much lower than those of raw biocrude oil (404 Da) and the oil product obtained using the other catalysts (522 and 389 Da). The substantial reduction in molecular weight results in the change in density of the oils after the upgrading treatments (table S8). Among all oils tested, raw biocrude oil has the highest density of 0.853 g/ml, and the upgraded oil using Fe-Mo_2_C/ZSM-5 has the lowest density of 0.783 g/ml.

### Prescreening results as a SAF precursor

**Fig. 3.**
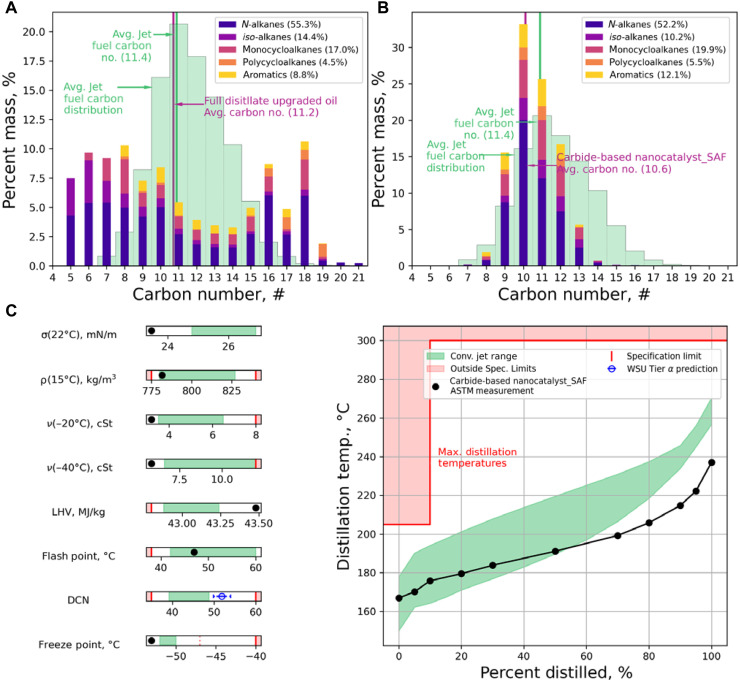
Analysis of upgraded fuel and its distillate cut as SAF precursor. Hydrocarbon type analysis, carbon distribution (colored bar), and average carbon number (magenta vertical line) of (**A**) full distillate fuel and (**B**) distilled SAF fraction. The average conventional jet fuel carbon distribution (green shaded region) and the average carbon number (green vertical line) are shown for references. (**C**) Tier beta property measurements for the SAF fraction. The conventional jet fuel experience range (green shaded region), specification limit (red line), and outside the specification limit (red shaded region) are plotted for reference.

The property of hydrocarbon in the product was analyzed on the full distillate range fuel using two-dimensional gas chromatography (GC × GC) using the standard methods described elsewhere (Fig. 3A) (*42*). The full distillate of upgraded biocrude oil ranges from C5 to C21, with an average carbon number of 11.2 close to that of the conventional jet fuel (11.4), and exhibits a bimodal distribution peaked around C8 and C18, consisting of *n*- and *iso-*alkanes (69.7%), cycloalkanes (21.5%), and aromatics (8.8%). While the average carbon number is similar to that of jet fuel, the bimodal carbon distribution needs further treatment to pass the ASTM D4054 evaluation. Thus, it is pragmatic to extract the most suitable fraction of the upgraded oil for SAF certification tests.

We obtained the target fraction from the full range biocrude oil by performing distillation, which was executed from 150° to 230°C, focusing on meeting the flash point and freezing point specification limits of the final fuel because these two properties are crucial to address aviation safety issues (*43*). The front end of distillation is determined to be 150°C, matching that of the conventional jet fuel to ensure a comparable flash point. The back end of the distillation, however, is truncated at 230°C, instead of 260°C for jet fuel, due to the concentrated *n*-alkanes in the obtained biocrude oil. Freezing point of fuels is typically dominated by molecules having the highest freezing points in oil (*44*), normally the heaviest hydrocarbon species. Higher carbon number *n*-alkanes generally have higher freezing points than their isomerized counterparts. The inclusion of C14 to C16 components in the SAF cut is detrimental to the increase of freezing point. Thus, we reduced the back end of the distillation range to ensure a proper freezing point. The yield of SAF precursors from the temperature range of 150° to 230°C is ~15 wt %.

Figure 3B illustrates the analysis of hydrocarbon types of the distilled SAF precursor. The average carbon number of the SAF fraction is 10.6 compared to 11.4 for conventional jet fuel. Compared to the full range oil, the SAF precursor has higher contents of cycloalkanes (25.4%) and aromatics (12.1%), with a distribution peaked around C10. Tier beta prescreening was conducted on the distilled SAF fraction, evaluating properties such as surface tension, density, kinematic viscosity, LHV, flash point, freezing point, and distillation curve. The derived cetane number (DCN) is predicted from estimation due to the volume limitation. These properties are critical for combustor figures of merits and operability limits (*17*). Aligning the SAF precursor within the specification limit can mitigate risks at the earliest stage of conversion technology development. As shown in Fig. 3C, our SAF fraction demonstrated excellent surface tension, smaller than 24 mN/m at 22°C, which is better than the experience range of conventional jet fuel. The viscosity at −20° and −40°C also outperformed the conventional jet fuel. The density of obtained SAF precursor is relatively low compared with the conventional fuel but still within the acceptable range. Low values in these properties enhance combustor operability limits and are attributed to the cutoff point of 230°C, which excludes heavier fractions (>C14). Energy density of this oil is much higher than the other cuts, as suggested by the LHV ~43.50 MJ/kg, which can be attributed to the high concentration of *n*- and *iso-*alkanes. The flash point of SAF precursor is well fitted within the experience range, suggesting a close resemblance with conventional jet fuel regarding the light components. The freezing point is superior because of the approach of excluding >C14 substances. Thus, an optimal distillation range was identified for this fuel to achieve a balance between SAF yield and control of specific properties, such as freezing point. Tier alpha property predictions indicate a high DCN value for the SAF fraction, which could result from the richness of *n*- and *iso*-alkane content. Overall, the obtained distillate cut reached the threshold for key physical properties set for SAF, indicating that Fe-Mo_2_C/ZSM-5 is an appropriate catalyst for converting food waste–derived biocrude oil to high-quality SAF product, and other conversion pathway and processing protocol need to be further developed.

Figure 3C summarizes the results of an overall assessment performed by evaluating the distillation curve, which contrasts against that of the conventional jet fuel. Our SAF precursor falls in the experience range up to ~60 wt %, with the heavy section slightly deviating from the target due to the lack of C14 to C16 components in the distillate to meet the freezing point specification, which is related to the high concentration of *n*-alkane (52.2%). From an economic perspective, the current conversion pathway is attractive because besides the SAF precursor, the C4 to C8 cut can be used for gasoline and the C14 to C21 cut as biodiesel. Heavier fraction (C22 and above) in the biocrude can be upgraded for marine fuel or lubricant. We noted that a multistep approach was previously developed using methane as a feedstock to produce *n*-paraffin SAF designed as a 10 vol % blend (*40*). In that process, instead of directly upgrading biocrude, methane was converted into mixed volatile C_2_ to C_8_ fatty acids (VFAs) via anaerobic digestion. Ketonization over ZrO_2_ and hydrodeoxygenation over Pt/Al_2_O_3_ were then carried out to convert the wet waste–derived VFAs into normal paraffins.

### Compositional and structural stability of Fe-Mo_2_C/ZSM-5 catalyst

**Fig. 4.**
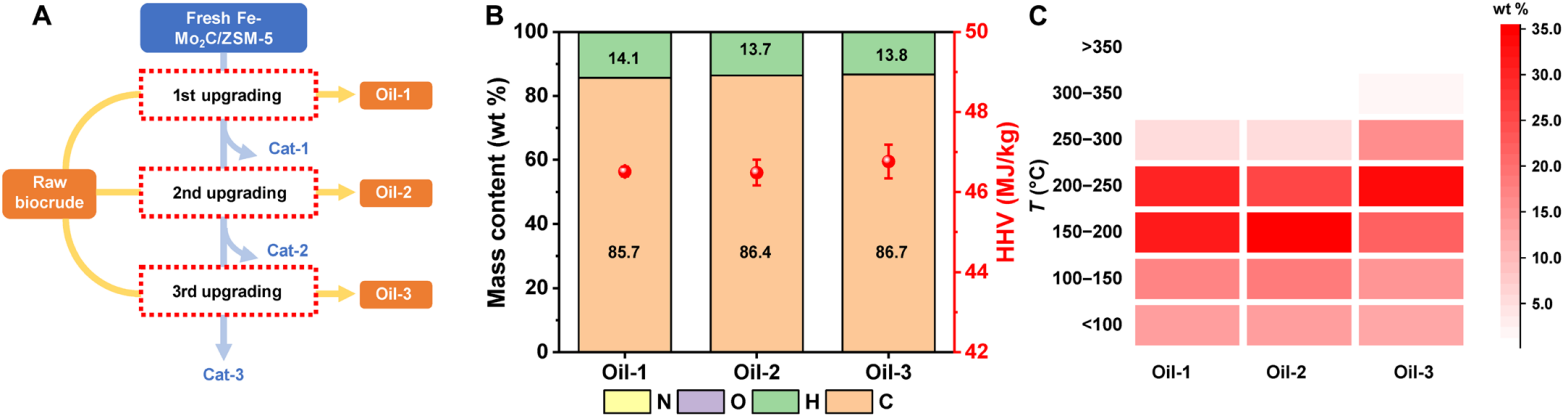
Fe-Mo_2_C/ZSM-5 catalysts were reused to upgrading the biocrude oils. (**A**) Illustration of the workflow, (**B**) mass content and HHV based on elemental analysis, and (**C**) boiling point distribution based on TGA.

We investigated the stability of Fe-Mo_2_C/ZSM-5 by reusing this catalyst for multiple runs (Fig. 4A). In a standard test, after the reaction, the solids including catalyst were separated from the upgraded oil by centrifugation, washed by acetone, and dried at 80°C in a vacuum oven. The Fe-Mo_2_C/ZSM-5 after the first upgrading test, which is denoted as Cat-1, was collected and separated from the upgraded oil (i.e., Oil-1) and used as the catalyst for the second upgrading. No reactivation of catalyst was needed, and all reaction conditions were kept the same for all the catalytic upgrading study (Fig. 4A). Figure 4B shows the HDO activity of reused Fe-Mo_2_C/ZSM-5 catalysts by analyzing the elemental contents of three upgraded oils. No oxygen was detected in all three upgraded oils, suggesting ~100% deoxygenation efficiency (table S9). The hydrogen content of upgraded oils decreased slightly from 14.08 wt % (Oil-1) to 13.74 wt % for Oil-2 and 13.85 wt % for Oil-3. The H/C molar ratio changed from 1.96 (Oil-1) to 1.90 for Oil-2 and 1.90 for Oil-3. The reused Fe-Mo_2_C/ZSM-5 catalysts (Cat-1 and Cat-2) were still active to produce upgraded oils with the HHV that is comparable to Jet A (~ 46.10 MJ/kg; table S4). Elemental analysis results indicate density of the three upgraded oils is consistent (0.779 to 0.783 g/ml; table S10). Both Oil-2 and Oil-3 exhibited higher carbon content. TGA results show the boiling point distributions for all thee upgraded oils are concentration in the range of 150° to 250°C (Fig. 4C; figs. S13, A and B; and table S11).

XRD and x-ray photoelectron spectroscopy (XPS) were used to examine the structures of Fe-Mo_2_C/ZSM-5 catalyst after each run of upgrading of biocrude oils. The XRD study shows β-Mo_2_C and ZSM-5 phases retained for all reused Fe-Mo_2_C/ZSM-5 catalysts (i.e., Cat-1, Cat-2, and Cat-3) and no identifiable coke formed (fig. S14). The morphology and particle size of post-upgrading Fe-Mo_2_C/ZSM-5 catalysts did not show major difference from that of the freshly prepared (fig. S15). Rietveld refinement analysis indicates the average size of Mo_2_C nanoparticles remained to be in the range of sub–3 nm (fig. S16 and table S12), which were also observed in the high-resolution electron microscopic images (fig. S17). Surface composition of the reused catalyst was investigated by comparing the valence state of Mo and C based on the XPS study (fig. S18 and table S13). The intensity of Mo(II) peaks for Cat-1 at 231.8 eV (3d_3/2_) and 228.6 eV (3d_5/2_) is comparable to that of fresh catalyst. Carbon peaks in the carbides were observed for both Cat-1 and fresh catalysts and no detectable change of surface Mo_2_C nanoparticles after the upgrading run, indicating good structural and compositional stability.

The XPS Fe 2p data show Fe^2+^ or Fe^3+^ 2p_1/2_ (~724 eV) and 2p_3/2_ (~710 eV) signals appeared in the Cat-1 sample besides the Fe^0^ 2p_3/2_ (~707 eV) in the Fe-Mo_2_C/ZSM-5 (fig. S18) (*45*, *46*). This observation indicates the nanocatalysts could be oxidized either during or after the upgrading process, suggesting that the structures of Fe species might change in the Fe-Mo_2_C/ZSM-5. We also performed TGA analysis of fresh Fe-Mo_2_C/ZSM-5 and Cat-1 to further study coke formation (fig S19). The mass loss curves for both samples show comparable curves in terms of the weight loss in the entire temperature range. In the temperature range between 270° and 400°C, Cat-1 exhibits larger derivative in weight gain than that of the fresh catalyst. These data suggest the oxidizable species in Cat-1 such as carbide and iron could take up oxygen faster than the freshly made catalysts, in agreement with the XPS Fe 2p data. Nevertheless, the Fe-Mo_2_C/ZSM-5 catalyst is readily reusable for oil upgrading, suggesting that the highly reducing condition of hydrodeoxygenation reaction is highly tolerable of initial structure changes, and catalysts can be regenerated in situ during the process.

## DISCUSSION

This work demonstrates the feasibility of catalytic upgrading of wet waste–derived biocrude oil into SAF precursors using zeolite-supported Mo_2_C nanocatalyst. We found that Fe-Mo_2_C/ZSM-5 catalyst exhibited excellent HDO activity to fully deoxygenate the biocrude oil produced from HTL process. We achieved a yield of 56 wt % for SAF precursor from pretreated biocrude, equivalent to a food waste to SAF production yield of 23 wt %, based on a yield of 59 wt % from food waste to biocrude and a loss of 30 wt % during the pretreatment of biocrude (*47*–*49*). The upgrade oil exhibits the HHV over 46.10 MJ/kg comparable to Jet A. This high performance can be attributed to the nanometer-sized Mo_2_C catalyst on Bronsted acid–based zeolitic supports, which remain compositionally and structurally stable in the stability tests. This work develops low-cost metal carbide catalysts and demonstrates their potential in converting biocrude oils, oleochemical-based fermentation products, and crop residue-derived biomass into SAF candidates.

## MATERIALS AND METHODS

### Chemical and materials

Ammonium molybdate (para) tetrahydrate [(NH_4_)_6_Mo_7_O_24_·4H_2_O, 99%, A13766] and the reference beta-phase molybdenum carbide (β-Mo_2_C, 99.5% metals basis, 012192.36) were purchased from Alfa Aesar. Iron (III) nitrate nonahydrate ( ≥98%; ACS reagent, 216828) was obtained from Sigma-Aldrich. Zeolite ZSM-5 (ammonium, 045880.22) and quartz wool (fine, 4 μm; 451040100) were obtained from Thermo Fisher Scientific. Methane [ultra-high purity (UHP)], hydrogen (UHP), argon (UHP), compressed air (breathing quality grade), and nitrogen were purchased from Airgas. Acetone (ACS grade, A18-4) was purchased from Fisher Chemical. Jet A sample was obtained from a local airport. Biocrude oil was produced from HTL via our laboratory-made continuous plug-flow pilot reactor using food waste as feedstock, as detailed in previous work (*47*–*49*). In summary, salad dressing waste (62% lipid, 30% carbohydrate, 2% protein, and 6% ash) was obtained from a local food processing plant and homogenized to a solids content of 20 wt %. The food waste was converted at 300°C and 11 MPa, with a retention time of 20 min, yielding 59 wt % biocrude by mass. Before upgrading, the HTL biocrude oil was washed and distilled to remove salt (mainly Na and K), excess water, and ash. In all experiments, deionized (DI) water (resistivity of 18.25 MΩ·cm at room temperature, Millipore) was used.

### Synthesis of Fe-Mo_2_C/ZSM-5

The metal precursor was prepared via the wet impregnation method. In a 100-ml flask, Fe(NO_3_)_3_·9H_2_O (171.6 mg), (NH_4_)_6_Mo_7_O_24_·4H_2_O (1.5 g), and ZSM-5 (1.0 g) were added to 20 ml of DI water. The mixture was heated at 80°C (250 rpm) for 8 hours in an oil bath using a hotplate stirrer (VWR 7x7 Ceramic Hotplate Stirrer, 97042-714). After water evaporated, the remaining mixture was further dried at 80°C in a vacuum oven (VWR Symphony Vacuum Oven, catalog no. 414004-582) overnight. The dried solids were collected and ground into a fine powder. The powder precursor (1.0 g) was loaded into a quartz tube, which was placed inside a vertical furnace. Quartz wool was used as a support for the powder precursor in a vertically placed tube. After the assembly, the quartz tube was purged with a flowing gas mixture of CH_4_/H_2_ (flow rate ratio: 8/32, 40 sccm in total). The subsequent carburization step was programmed to firstly reach 300°C at a rate of 4°C/min and then to 600°C at 1°C/min. The temperature was then kept at 600°C for 2 hours. After the furnace was naturally cooled to room temperature, the quartz tube was purged by N_2_ for 10 min (90 sccm). The N_2_-protected solids were carefully transferred from the quartz tube to a test tube (length > 10 cm) filled with Ar to avoid exposure of catalyst to air. The test tube was then sealed with Parafilm for future usage in oil upgrading. The time of storage was typically <24 hours. Considering the pyrophoric nature of metal carbide nanoparticles, we synthesized the catalyst right before the upgrading tests. For material characterization, the as-made catalysts were passivated in 2% O_2_ (a mixture of 10 sccm compressed air and 90 sccm N_2_) for 1 hour after the purge by N_2_ in the furnace for 10 min. The sample can be safely removed from the quartz tube. [Caution: Freshly made molybdenum carbide nanoparticles are highly reactive if they are not passivated and ignite upon their exposure to ambient air.]

### Structural and compositional characterization of catalysts

XRD patterns were obtained using Rigaku Miniflex 600 diffractometer equipped with Cu Kα x-ray source (λ =1.54056 Å). The size analysis based on XRD patterns were conducted using a profile fitting software, TOPAS, provided by Bruker Corporation. This software was used to obtain the volume-weighted mean crystallite sizes (LVol-IB) calculated from Scherrer equation based on Rietveld refinement results. SEM images were obtained from Hitachi S-4800 SEM. TEM micrographs were from JEOL 2100 Cryo microscope. High-resolution STEM and EDS studies were conducted using FEI Themis Z advanced probe aberration corrected analytical scanning/transmission electron microscope. The mass contents of Fe and Mo in the samples were determined by ICP-OES using a PerkinElmer Optima 8300 system. XPS data were obtained using Kratos Axis Supra+ Photoelectron spectrometer, and the corresponding data analysis was performed using CasaXPS software. The identification, deconvolution, and assignment of XPS peaks were based on the references provided in an XPS handbook (*45*, *46*). The XPS data were analyzed after the calibration using the adventitious carbon peak at binding energy of 284.80 eV. The deconvolution of overlapped peaks in Mo 3d spectra was carried out using the principle of spin-orbit splitting ratio for 3d spectrum, i.e., 2:3 for the signals from 3d_3/2_ and 3d_5/2_, respectively.

### Catalytic upgrading tests of biocrude oils

Upgrading tests of biocrude oils were carried out using a 100-ml Parr 4598 reactor with a Parr 4848 reactor controller. The biocrude oil (10 ml) was added to an Ar-protected, freshly prepared, non-passivated Fe-Mo_2_C/ZSM-5 catalyst (~0.5 g) in the test tube. The mixture was stirred and transferred to a 100-ml stainless steel reaction chamber, which was then assembled in the Parr reactor. The reactor was purged with ~500-psi H_2_ three times before it was pressurized to 1500-psi H_2_ (~20°C), followed by a heat treatment at 400°C for 2 hours. The heating rate was set at ~25°C/min. The maximum pressure in the reactor was ~2200 psi at 400°C. After the reaction, the reactor was quenched in cold water for 10 min. The upgraded oils and used catalysts were collected and separated by centrifugation at 9000 rpm for 10 min (Beckman Coulter Inc., Allegra X-30 Series). The used catalysts were washed with acetone three times before drying in the vacuum oven overnight. For control experiments using commercial Mo_2_C or ZSM-5, all reaction conditions were kept the same other than the catalysts used. The weight of individual components, other than the total amount of catalysts, was also kept the same, i.e., ~0.5 g of Fe-Mo_2_C/ZSM-5 was replaced with ~0.23 g of commercial Mo_2_C or ~0.27 g of commercial ZSM-5 in the control upgrading tests, according to the theoretical value based on feeding ratio (table S1). When performance was tested with the used Fe-Mo_2_C/ZSM-5, all reaction conditions were kept the same other than the catalyst. The amount of used Fe-Mo_2_C/ZSM-5 for the upgrading test is 0.5 g. No pretreatment or reactivation of used catalysts was conducted.

### Characterization of biocrude oils and analytical methods

The elemental content was determined using CE440 CHN analyzer from Exeter Analytical. The oxygen content was calculated by the difference, i.e., 100-∑(C+H+N) (wt %). The HHV of biocrude oils was calculated using Dulong’s formula (*50*). The boiling point distribution of oil samples was obtained using a Q50 Thermogravimetric Analyzer from TA Instruments. The sample was placed in a heating chamber purged with N_2_, which could mimic the distillation processing. The samples were heated from room temperature to 600°C at a rate of 20°C/min in N_2_ flow at atmospheric pressure. The TGA data analysis was performed using Universal Analysis 2000 software, provided by TA Instruments, Waters LLC. The distribution of molecular weight of the compounds in biocrude oils was studied by performing MALDI-TOF-MS using Bruker Autoflex Speed LRF instrument. The scan range of mass spectra is between 50 and 1500 Da. The *M*_n_, *M*_m_, and polydispersity index (*I*) were calculated using the following equations

$$M_{n}=\sum \left(M_{i}\times \frac{N_{i}}{\sum N_{j}}\right)=\frac{\sum \left(M_{i}N_{i}\right)}{\sum N_{j}}$$ (1)

$$M_{m}=\sum \left[M_{i}\times \frac{M_{i}N_{i}}{\sum \left(M_{j}N_{j}\right)}\right]=\frac{\sum \left(M_{i}^{2}N_{i}\right)}{\sum \left(M_{j}N_{j}\right)}$$ (2)

$$I=\frac{M_{m}}{M_{n}}$$ (3)

where $M_{i}$ and $N_{i}$ are the mass (in unit of Da) and background-corrected abundance of the ith oligomer, respectively. The background baseline is determined on the basis of averaged noise level of the mass spectra. The polydispersity index (*I*) measures the dispersion of molecular weights of chemical compounds in the oil, i.e., *I* of one suggests a homogenous weight distribution, and larger *I* values (i.e., *I* > 1) imply widely dispersed weight distribution.

Hydrocarbon type analysis was performed using two-dimensional gas chromatography (GC × GC), of which detailed setup can be found in a previous work (*42*). The oil went through distillation using a Micro Spinning Band from B/R Instrument, which offers excellent theoretical plates and minimal dead volume. The distillate cut was executed from 150° to 230°C based on the flash point and freezing point specification limits (*43*).

## References

[1] V. Undavalli, O. B. Gbadamosi Olatunde, R. Boylu, C. Wei, J. Haeker, J. Hamilton, B. Khandelwal, Recent advancements in sustainable aviation fuels. Prog. Aeronaut. Sci. 136, 100876 (2023).

[2] K. S. Ng, D. Farooq, A. Yang, Global biorenewable development strategies for sustainable aviation fuel production. Renew. Sustain. Energy Rev. 150, 111502 (2021).

[3] D. R. Vardon, B. J. Sherbacow, K. Guan, J. S. Heyne, Z. Abdullah, Realizing “net-zero-carbon” sustainable aviation fuel. Joule 6, 16–21 (2022).

[4] The Air Transport Action Group, “Waypoint 2050” (2021).

[5] C. Gutiérrez-Antonio, F. I. Gómez-Castro, J. A. de Lira-Flores, S. Hernández, A review on the production processes of renewable jet fuel. Renew. Sustain. Energy Rev. 79, 709–729 (2017).

[6] M. L. Stone, M. S. Webber, W. P. Mounfield, D. C. Bell, E. Christensen, A. R. C. Morais, Y. Li, E. M. Anderson, J. S. Heyne, G. T. Beckham, Y. Román-Leshkov, Continuous hydrodeoxygenation of lignin to jet-range aromatic hydrocarbons. Joule 6, 2324–2337 (2022).

[7] M. M. Abu-Omar, K. Barta, G. T. Beckham, J. S. Luterbacher, J. Ralph, R. Rinaldi, Y. Román-Leshkov, J. S. M. Samec, B. F. Sels, F. Wang, Guidelines for performing lignin-first biorefining. Energ. Environ. Sci. 14, 262–292 (2021).

[8] S. Geleynse, K. Brandt, M. Garcia-Perez, M. Wolcott, X. Zhang, The alcohol-to-jet conversion pathway for drop-in biofuels: Techno-economic evaluation. ChemSusChem 11, 3728–3741 (2018). 30212605 10.1002/cssc.201801690

[9] S. Subramaniam, D. M. Santosa, C. Brady, M. Swita, K. K. Ramasamy, M. R. Thorson, Extended catalyst lifetime testing for HTL biocrude hydrotreating to produce fuel blendstocks from wet wastes. ACS Sustainable Chem. Eng. 9, 12825–12832 (2021).

[10] D. V. Cabrera, R. A. Labatut, Outlook and challenges for recovering energy and water from complex organic waste using hydrothermal liquefaction. Sustainable Energy Fuels 5, 2201–2227 (2021).

[11] Z. Wang, J. Watson, T. Wang, S. Yi, B. Si, Y. Zhang, Enhancing energy recovery via two stage co-fermentation of hydrothermal liquefaction aqueous phase and crude glycerol. Energ. Conver. Manage. 231, 113855 (2021).

[12] L. B. Silva Thomsen, K. Anastasakis, P. Biller, Wet oxidation of aqueous phase from hydrothermal liquefaction of sewage sludge. Water Res. 209, 117863 (2022). 34844067 10.1016/j.watres.2021.117863

[13] D. J. Cronin, S. Subramaniam, C. Brady, A. Cooper, Z. Yang, J. Heyne, C. Drennan, K. K. Ramasamy, M. R. Thorson, Sustainable aviation fuel from hydrothermal liquefaction of wet wastes. Energies 15, 1306 (2022).

[14] W.-T. Chen, Y. Zhang, T. H. Lee, Z. Wu, B. Si, C.-F. F. Lee, A. Lin, B. K. Sharma, Renewable diesel blendstocks produced by hydrothermal liquefaction of wet biowaste. Nat. Sustain. 1, 702–710 (2018).

[15] W.-T. Chen, Y. Zhang, J. Zhang, L. Schideman, G. Yu, P. Zhang, M. Minarick, Co-liquefaction of swine manure and mixed-culture algal biomass from a wastewater treatment system to produce bio-crude oil. Appl. Energy 128, 209–216 (2014). 10.1016/j.biortech.2013.10.111 24287452

[16] J. Heyne, B. Rauch, P. Le Clercq, M. Colket, Sustainable aviation fuel prescreening tools and procedures. Fuel 290, 120004 (2021).

[17] M. Colket, J. Heyne, *Fuel Effects on Operability of Aircraft Gas Turbine Combustors* (The American Institute of Aeronautics and Astronautics Inc., 2021).

[18] D. Lin, Z. Mao, J. Shang, H. Zhu, T. Liu, Y. Wu, H. Z. Li, C. Peng, X. Feng, Catalyst design strategies for deoxygenation of vegetable oils to produce second-generation biodiesel. Ind. Eng. Chem. Res. 62, 12462–12481 (2023).

[19] S. Ding, C. M. A. Parlett, X. Fan, Recent developments in multifunctional catalysts for fatty acid hydrodeoxygenation as a route towards biofuels. Mol. Catal. 523, 111492 (2022).

[20] L. H. Bennett, J. R. Cuthill, A. J. McAlister, N. E. Erickson, R. E. Watson, Electronic and catalytic properties of tungsten Carbide. Science 187, 858–859 (1975). 1114333 10.1126/science.1114333

[21] J. Wan, Q. Liu, T. Wang, H. Yuan, P. Zhang, X. Gu, Theoretical investigation of platinum-like catalysts of molybdenum carbides for hydrogen evolution reaction. Solid State Commun. 284-286, 25–30 (2018).

[22] S. A. W. Hollak, R. W. Gosselink, D. S. van Es, J. H. Bitter, Comparison of tungsten and molybdenum carbide catalysts for the hydrodeoxygenation of oleic acid. ACS Catal. 3, 2837–2844 (2013).

[23] H. Guo, J. Zhao, Y. Chen, X. Lu, Y. Yang, C. Ding, L. Wu, L. Tan, J. Long, G. Yang, Y. Tang, N. Tsubaki, X. Gu, Mechanistic insights into hydrodeoxygenation of lignin derivatives over Ni single atoms supported on Mo_2_C. ACS Catal. 14, 703–717 (2024).

[24] S. C. Ammal, A. Heyden, Active site identification for glycerol hydrodeoxygenation over the oxygen modified molybdenum carbide surface. ACS Catal. 13, 7499–7513 (2023).

[25] T. Iida, M. Shetty, K. Murugappan, Z. Wang, K. Ohara, T. Wakihara, Y. Román-Leshkov, Encapsulation of molybdenum carbide nanoclusters inside zeolite micropores enables synergistic bifunctional catalysis for anisole hydrodeoxygenation. ACS Catal. 7, 8147–8151 (2017).

[26] X. Du, R. Zhang, D. Li, C. Hu, H. Garcia, Molybdenum carbide as catalyst in biomass derivatives conversion. J. Energy Chem. 73, 68–87 (2022).

[27] X. Chen, X. Chen, C. Li, C. Liang, Engineering the structural formula of N-doped molybdenum carbide nanowires for the deoxygenation of palmitic acid. Sustain. Energy Fuels 4, 2370–2379 (2020).

[28] G. T. Wurzler, V. T. da Silva, D. de Almeida Azevedo, A. S. Ana da Silva, F. B. Noronha, Integrating bio-oil and carbohydrate valorization on the fractionation of sugarcane bagasse via Organosolv process using Mo_2_C-based catalysts. Fuel Process. Technol. 230, 107208 (2022).

[29] C. Mukarakate, K. Iisa, S. E. Habas, K. A. Orton, M. Xu, C. Nash, Q. Wu, R. M. Happs, R. J. French, A. Kumar, E. M. Miller, M. R. Nimlos, J. A. Schaidle, Accelerating catalyst development for biofuel production through multiscale catalytic fast pyrolysis of biomass over Mo_2_C. Chem. Catal. 2, 1819–1831 (2022).

[30] M. A. Machado, S. He, T. E. Davies, K. Seshan, V. Teixeira da Silva, Renewable fuel production from hydropyrolysis of residual biomass using molybdenum carbide-based catalysts: An analytical Py-GC/MS investigation. Catal. Today 302, 161–168 (2018).

[31] F. Feng, L. Wang, X. Zhang, Q. Wang, Self-pillared ZSM-5-supported Ni nanoparticles as an efficient catalyst for upgrading oleic acid to aviation-fuel-range-alkanes. Ind. Eng. Chem. Res. 58, 13112–13121 (2019).

[32] M. Limlamthong, A. C. K. Yip, Recent advances in zeolite-encapsulated metal catalysts: A suitable catalyst design for catalytic biomass conversion. Bioresour. Technol. 297, 122488 (2020). 31796381 10.1016/j.biortech.2019.122488

[33] J. Jae, G. A. Tompsett, A. J. Foster, K. D. Hammond, S. M. Auerbach, R. F. Lobo, G. W. Huber, Investigation into the shape selectivity of zeolite catalysts for biomass conversion. J. Catal. 279, 257–268 (2011).

[34] Y. Wang, T. Li, C. Li, J. Lu, C. Dai, F. Subhan, P. Bai, H. Sun, R. Feng, Z. Yan, One-pot green synthesis of Fe-ZSM-5 zeolite containing framework heteroatoms via dry gel conversion for enhanced propylene selectivity of catalytic cracking catalyst. J. Mater. Sci. 56, 18050–18060 (2021).

[35] A. Kurbanova, D. Zákutná, K. Gołąbek, J. Hraníček, A. I. Dugulan, P. Diddams, M.-F. Hsieh, N. Bats, J. Přech, Fe-ZSM-5 outperforms Al-ZSM-5 in paraffin cracking by increasing the olefinicity of C3-C4 products. Chem. Eng. J. 499, 156032 (2024).

[36] M. Sedighi, K. Keyvanloo, J. Towfighi, Kinetic study of steam catalytic cracking of naphtha on a Fe/ZSM-5 catalyst. Fuel 109, 432–438 (2013).

[37] S. Rahimi, M. Rostamizadeh, Novel Fe/B-ZSM-5 nanocatalyst development for catalytic cracking of plastic to valuable products. J. Taiwan Inst. Chem. Eng. 118, 131–139 (2021).

[38] J. Li, X. Wang, X. Tang, M. Zhang, X. Zheng, C. Wang, Z. Tang, Upgrading of heavy oil by thermal treatment in the presence of alkali-treated Fe/ZSM-5, glycerol, and biomass. Fuel Process. Technol. 188, 137–145 (2019).

[39] W. Feng, Z. Xiao, B. Chen, Y. Pi, C. Hu, W. Zhang, Q. Meng, T. Wang, In situ confinement of ultrasmall Cu nanoparticles within silicalite-1 zeolite for catalytic reforming of methanol to hydrogen. Int. J. Hydrogen Energy 61, 113–124 (2024).

[40] N. A. Huq, G. R. Hafenstine, X. Huo, H. Nguyen, S. M. Tifft, D. R. Conklin, D. Stück, J. Stunkel, Z. Yang, J. S. Heyne, M. R. Wiatrowski, Y. Zhang, L. Tao, J. Zhu, C. S. McEnally, E. D. Christensen, C. Hays, K. M. Van Allsburg, K. A. Unocic, H. M. Meyer, Z. Abdullah, D. R. Vardon, Toward net-zero sustainable aviation fuel with wet waste–derived volatile fatty acids. Proc. Natl. Acad. Sci. U.S.A. 118, e2023008118 (2021). 33723013 10.1073/pnas.2023008118 PMC8020759

[41] J. G. Speight, *Handbook of petroleum product analysis* (Wiley, 2014).

[42] Z. Yang, S. Kosir, R. Stachler, L. Shafer, C. Anderson, J. S. Heyne, A GC×GC Tier α combustor operability prescreening method for sustainable aviation fuel candidates. Fuel 292, 120345 (2021).

[43] Z. Yang, R. C. Boehm, D. C. Bell, J. S. Heyne, Maximizing sustainable aviation fuel usage through optimization of distillation cut points and blending. Fuel 353, 129136 (2023).

[44] R. C. Boehm, A. A. Coburn, Z. Yang, C. T. Wanstall, J. S. Heyne, Blend prediction model for the freeze point of jet fuel range hydrocarbons. Energy Fuel 36, 12046–12053 (2022).

[45] J. F. Moulder, W. F. Stickle, P. E. Sobol, K. D. Bomben, *Handbook of X-Ray Photoelectron Spectroscopy* (PerkinElmer, 1992).

[46] X. Teng, D. Black, N. J. Watkins, Y. Gao, H. Yang, Platinum-maghemite core-shell nanoparticles using a sequential synthesis. Nano Lett. 3, 261–264 (2003).

[47] A. Aierzhati, J. Watson, B. Si, M. Stablein, T. Wang, Y. Zhang, Development of a mobile, pilot scale hydrothermal liquefaction reactor: Food waste conversion product analysis and techno-economic assessment. Energy Convers. Manage.: X 10, 100076 (2021).

[48] S. Summers, A. Valentine, Z. Wang, Y. Zhang, Pilot-scale continuous plug-flow hydrothermal liquefaction of food waste for biocrude production. Ind. Eng. Chem. Res. 62, 12174–12182 (2023).

[49] S. Summers, S. Yang, Z. Wang, B. Si, H. Kawale, Y. Zhang, Multi-stage pretreatment of hydrothermal liquefaction biocrude oil as a precursor for sustainable aviation fuel production. Fuel Process. Technol. 263, 108118 (2024).

[50] H. Li, Z. Zhu, J. Lu, J. Watson, D. Kong, K. Wang, Y. Zhang, Z. Liu, Establishment and performance of a plug-flow continuous hydrothermal reactor for biocrude oil production. Fuel 280, 118605 (2020).

