# Supplementary material for: Upgrading biocrude oil into sustainable aviation fuel using zeolite-supported iron-molybdenum carbide nanocatalysts
Source: Sci Adv. 2025 Jun 27;11(26):eadu5777. doi: 10.1126/sciadv.adu5777 (PMC12204169; doi:10.1126/sciadv.adu5777)
Supplement: Supplementary file 1 — Figs. S1 to S19 Tables S1 to S13 [file sciadv.adu5777_sm.pdf]

Science Advances
AAAS

Supplementary Materials for

Upgrading biocrude oil into sustainable aviation fuel using zeolite-supported iron-molybdenum carbide nanocatalysts

Siying Yu *et al.*

Corresponding author: Hong Yang, hy66@illinois.edu; Yuanhui Zhang, yzhang1@illinois.edu

Sci. Adv. **11**, eadu5777 (2025)
DOI: 10.1126/sciadv.adu5777

This PDF file includes:

Figs. S1 to S19
Tables S1 to S13

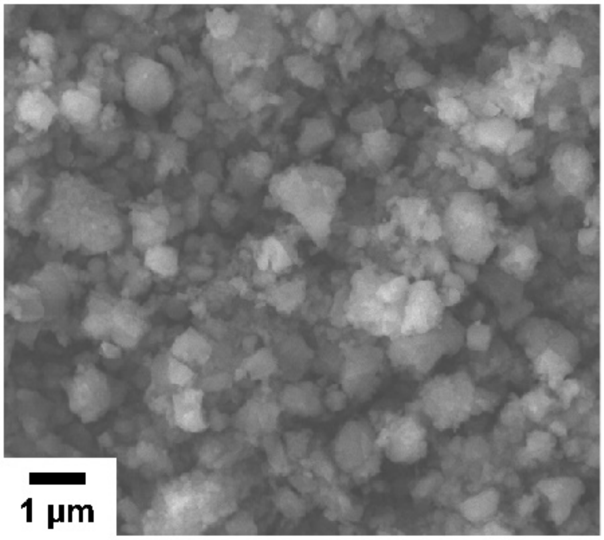

Fig. S1. Scanning electron microscopy (SEM) study of the zeolite support used in this study. SEM image of the commercial ZSM-5.

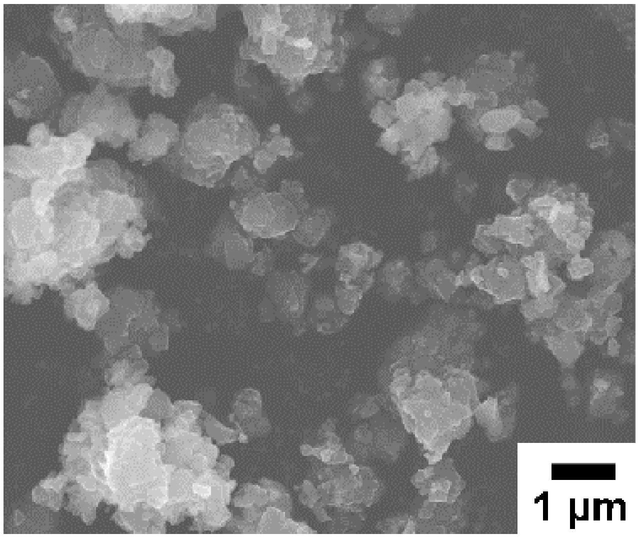

Fig. S2. SEM characterization of the as-made nanocatalysts used in this study. Low-mag SEM image of the freshly prepared Fe-Mo_2C/ZSM-5 catalyst.

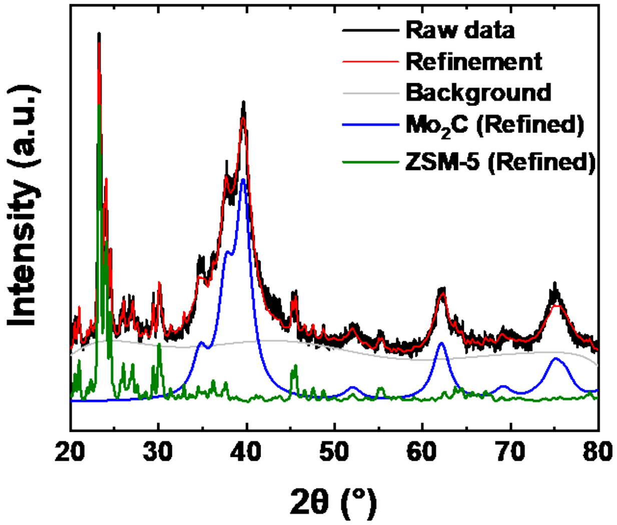

Fig. S3. X-ray diffraction (XRD) characterization and simulation of the Fe-Mo2C/ZSM-5 catalyst. Rietveld refinement results of XRD pattern of the Fe-Mo2C/ZSM-5 catalyst. The obtained XRD pattern (black curve) is contrasted with the fitting envelope (red curve), which consists of the contributions from Mo2C (blue curve) and ZSM-5 (green curve). The crystallographic information file (cif) used in the refinement can be found in Crystallography Open Database (COD). The COD ID is 5910009 for Mo2C and 1505106 for ZSM-5.

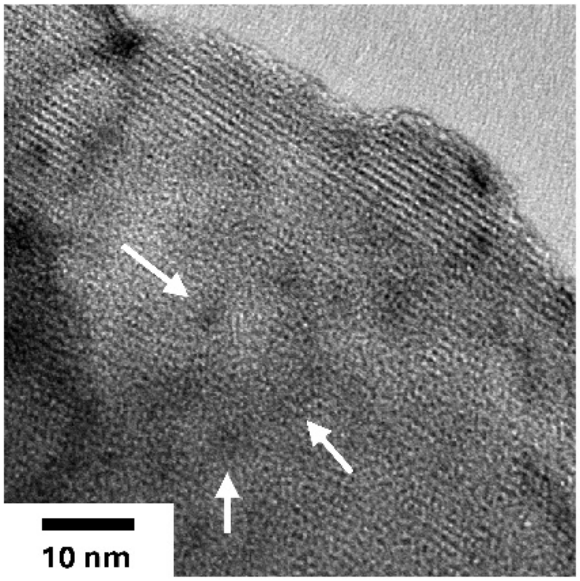

Fig. S4. Transmission electron microscopy (TEM) study of the Fe-Mo_2C/ZSM-5 nanocatalysts. TEM micrograph of the freshly prepared ~~and passivated~~ Fe-Mo_2C/ZSM-5 catalyst. The light grey area represents the ZSM-5 support while the dark areas pointed by the white arrows are the likely position of Mo_2C nanoparticles.

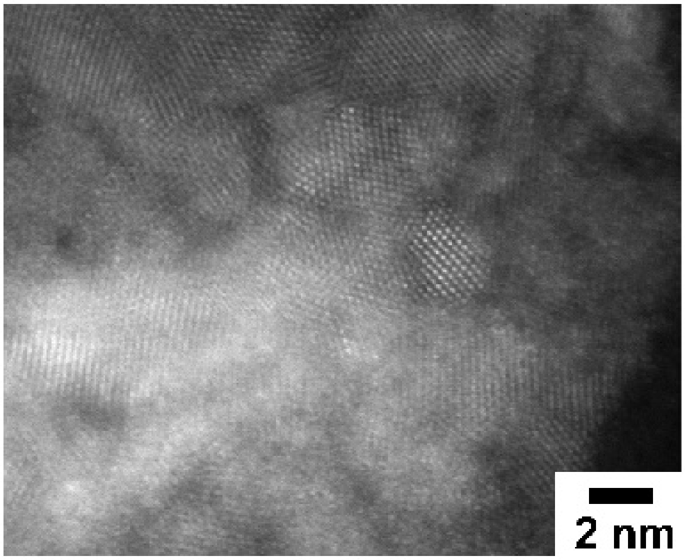

Fig. S5. Dark field scanning transmission electron microscopy (STEM) study of the Fe-Mo_2C/ZSM-5 nanocatalysts. Representative dark-field STEM micrograph shows the as-prepared Fe-Mo_2C/ZSM-5 catalysts contain fine, crystalline Mo_2C nanoparticles.

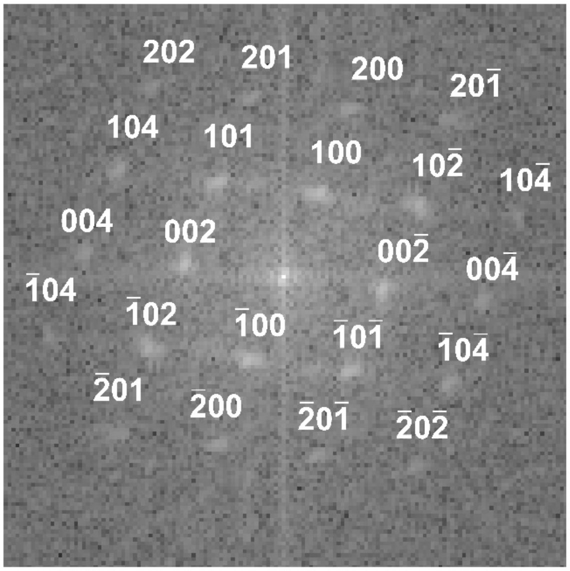

Fig. S6. Fast Fourier transform (FFT) analysis of carbide catalyst nanoparticles. The diffraction pattern of Mo_2C lattices in **Fig. 2c** are analyzed by the FFT method.

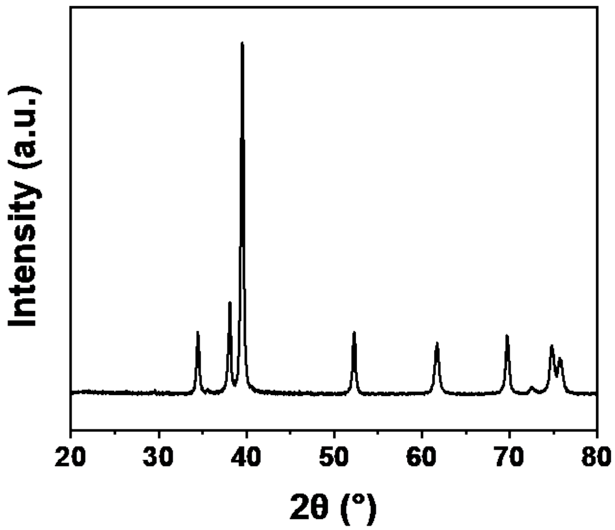

Fig. S7. XRD pattern of the commercial Mo_2C. The XRD study indicates the reference catalyst is made of a highly crystalline material of beta-Mo_2C**.**

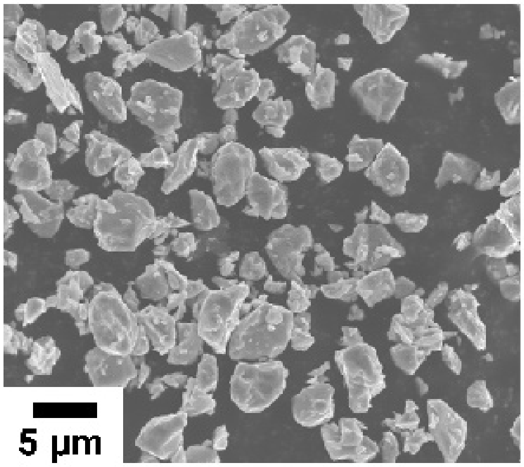

Fig. S8. SEM image of the commercial Mo_2C particles. The SEM study shows these metal carbide particles exhibit different crystalline morphologies and are in a size ranging up to a few micrometers.

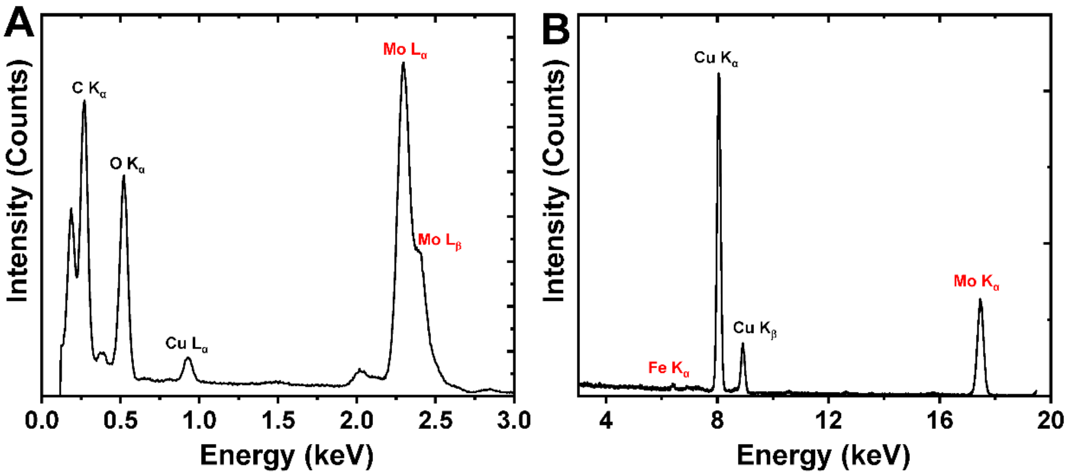

Fig. S9. Metal elements in the Fe-Mo_2C/ZSM-5 catalysts were analyzed by the energy dispersive X-ray spectroscopy (EDS). Dark-field STEM-EDS spectra of the as-prepared Fe-Mo_2C/ZSM-5 catalyst in the range of (**A**) 0~3 keV and (**B**) 3~20 keV. The carbon and copper signals were from the carbon-coated copper TEM grids.

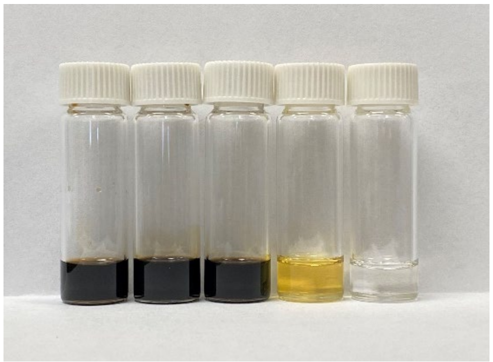

Fig. S10. Photo of upgraded oils obtained from different reaction conditions. From left to right: the raw biocrude oil (Raw); the upgraded oil without catalyst (No Cat.); the upgraded oil using the commercial Mo_2C (Comm.); the upgraded oil using the freshly prepared catalyst (Fe-Mo_2C/ZSM-5); and Jet A.

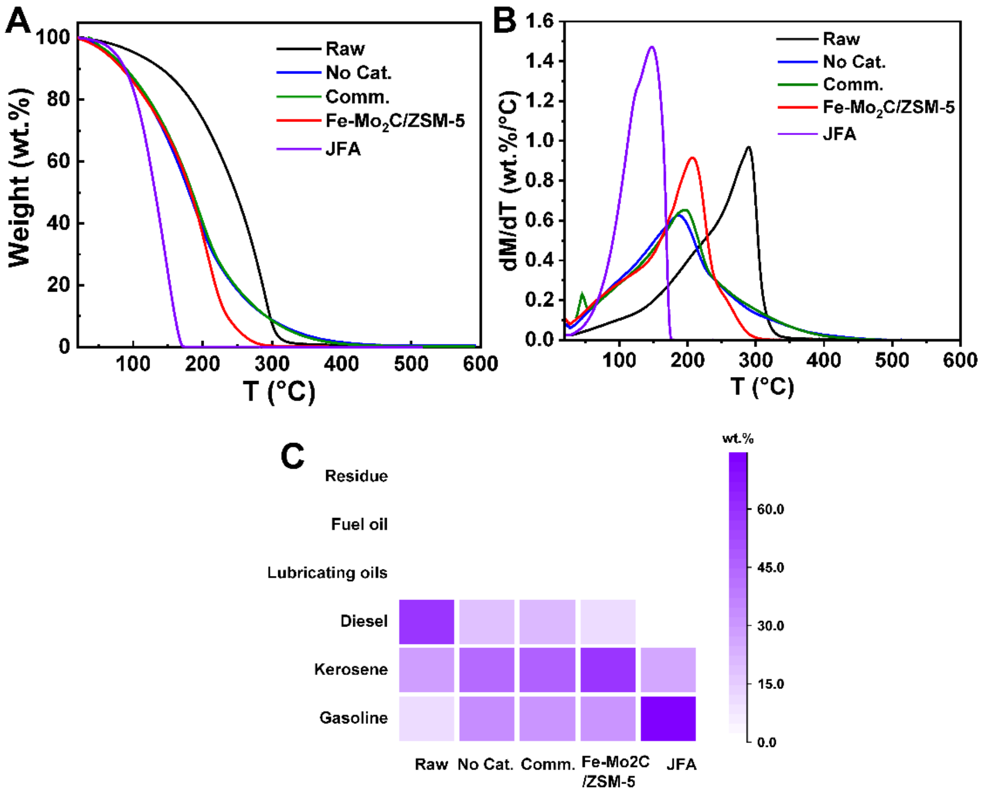

Fig. S11. Thermogravimetric analysis (TGA) results of the raw and upgraded oils. (**A**) Weight percentage curves versus temperature. (**B**) The derivative of weight loss (wt.%) versus temperature (°C). (**C**) Weight distributions regarding oil types calculated from the boiling point distribution. Data of Jet A (JFA) is included as reference.

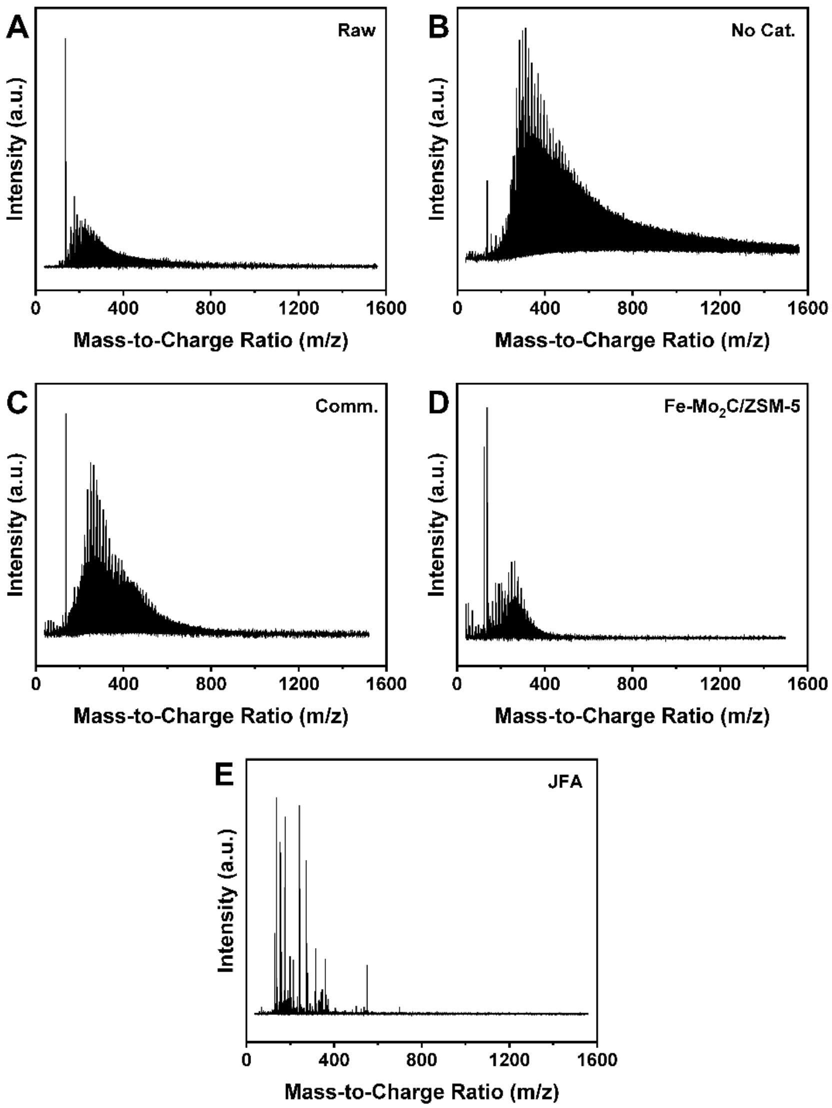

Fig. S12. Mass spectrometry (MS) study of molecular weight distribution of the upgraded oils and Jet A reference. MALDI-TOF-MS data of (A) untreated raw biocrude oil, and those treated (B) without catalyst (No Cat.) and with (C) commercial Mo_2C (Comm.) or (D) freshly prepared Fe-Mo_2C/ ZSM-5 catalyst. (E) Data of Jet A is included as reference.

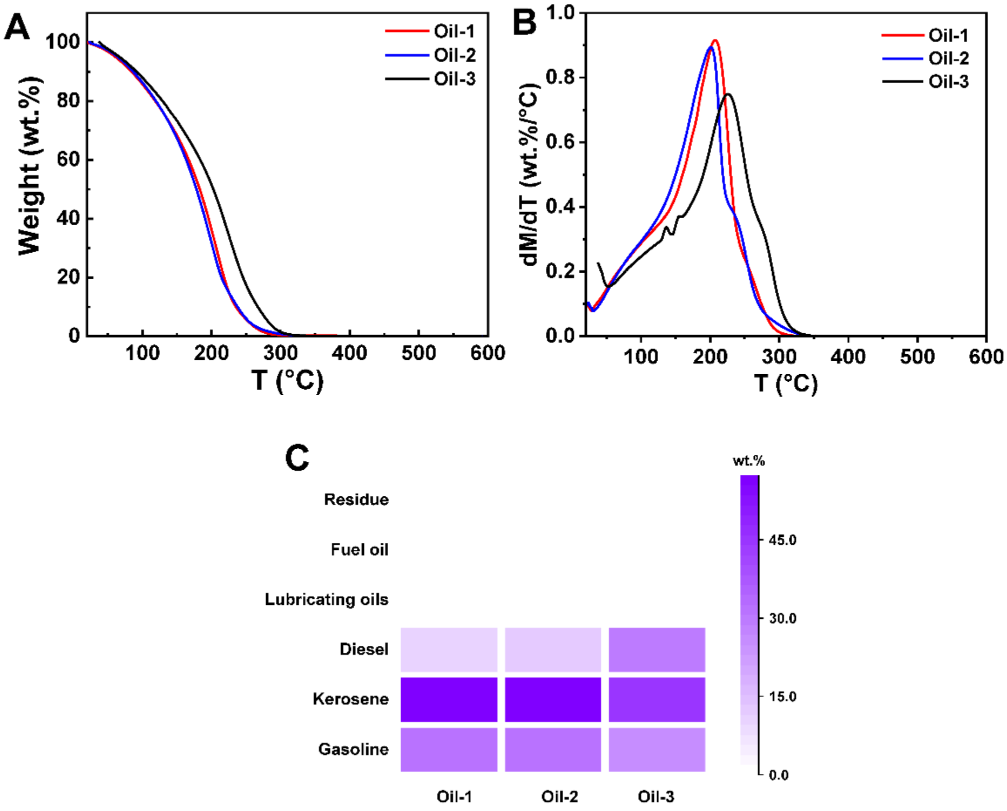

Fig. S13. TGA of upgraded oils using freshly prepared or reused Fe-Mo2C/ZSM-5 catalysts. (**A**) Weight percentage curves versus temperature, (**B**) Derivatives of weight loss (wt.%) versus temperature (°C), and (**C**) weight distributions based on oil types calculated from the boiling point distribution.

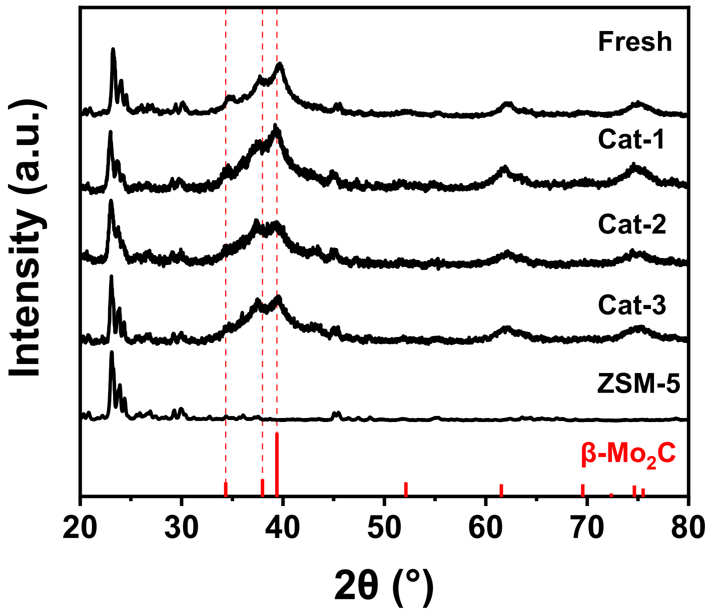

Fig. S14. XRD characterizations of the fresh and reused catalysts. The XRD patterns of the fresh and three reused catalysts and commercial ZSM-5 are compared, showing all exhibit the crystal phase of β-Mo_2C. The standard pattern of β-Mo_2C is listed (red color) as the reference.

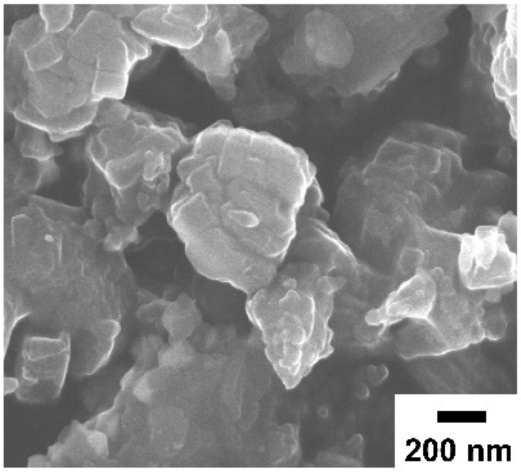

Fig. S15. Representative SEM image of the post-upgrading Fe-Mo_2C/ZSM-5. The SEM image of Cat-1 shows the typical crystalline morphologies of ZMS-5 and the nanoparticle carbides on the zeolite support.

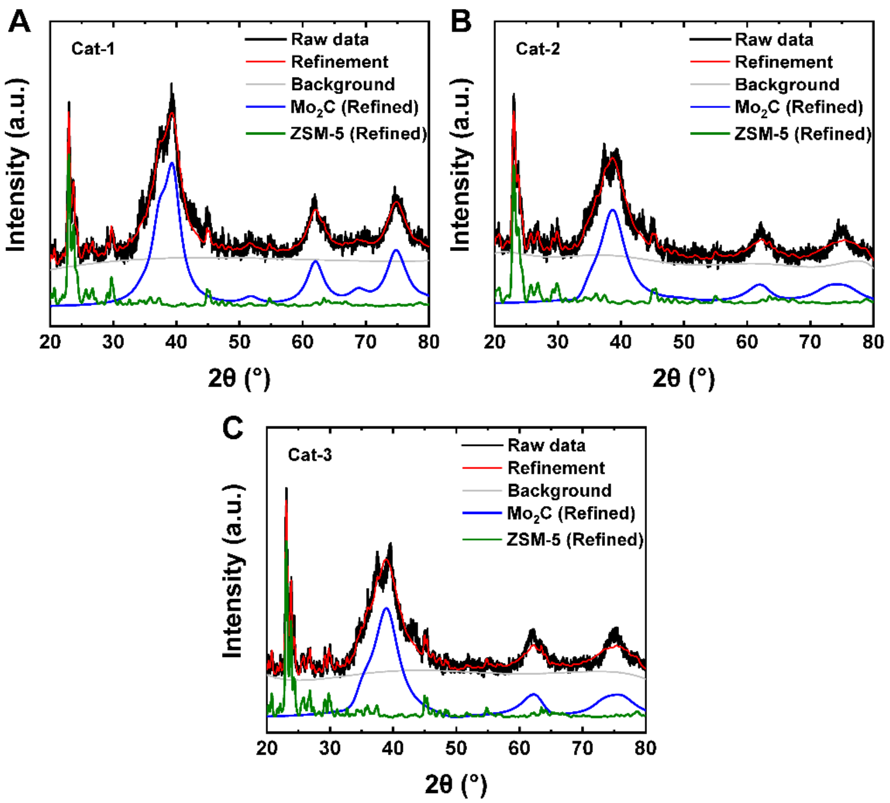

Fig. S16. Rietveld refinement analysis of XRD patterns for the three reused Fe-Mo_2C/ZSM-5 catalysts. The refinement results indicate all three catalysts contains Mo_2C and ZSM-5. The broad XRD peaks for Mo_2C suggest the metal carbide retains its nanocrystalline feature.

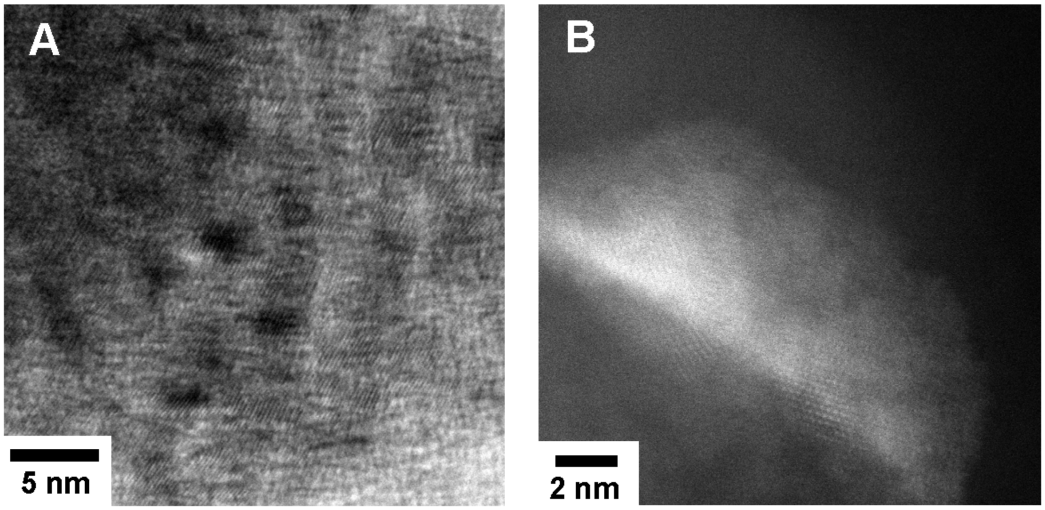

Fig. S17. TEM micrographs of reused Fe-Mo_2C/ZSM-5 catalysts. (**A**) TEM and (**B**) high angle annular dark field (HAADF)-STEM micrographs of the Fe-Mo_2C/ZSM-5 catalyst after the upgrading test (Cat-1).

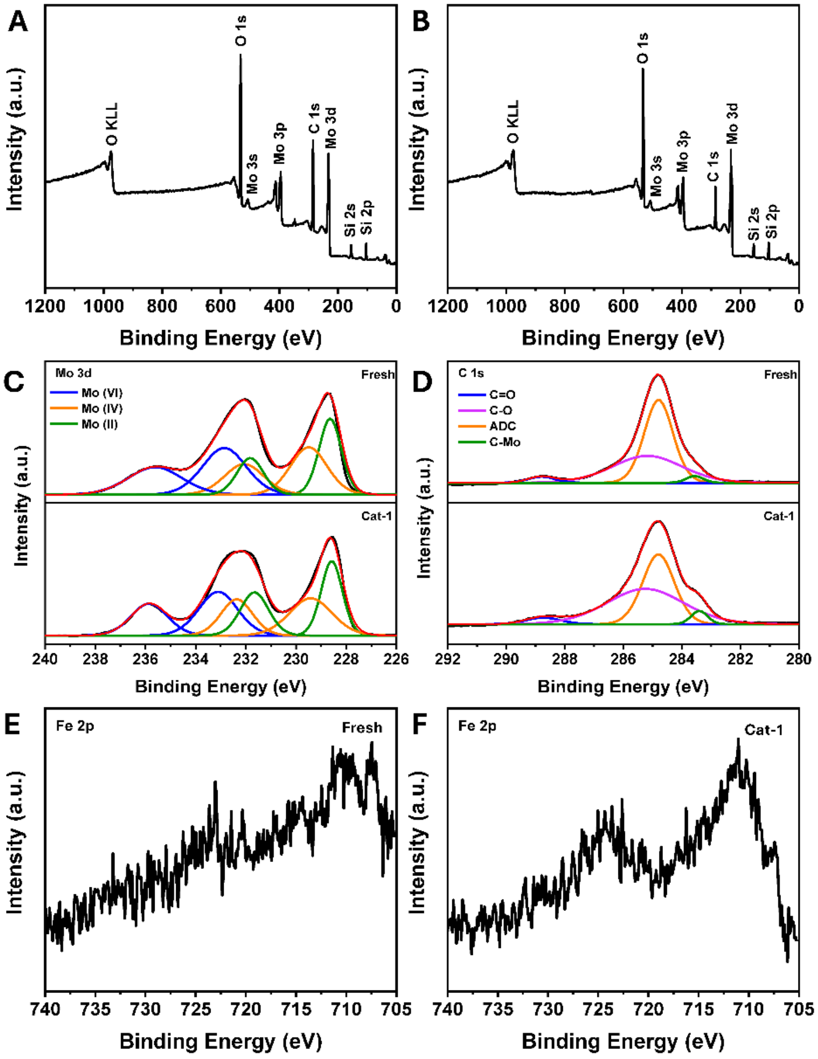

Fig. S18. XPS analysis of freshly prepared and reused Fe-Mo_2C/ZSM-5 catalysts. XPS survey scans of (**A**) freshly prepared Fe-Mo_2C/ZSM-5 catalyst and (**B**) Cat-1, respectively. (**C**) Mo 3d spectra, (**D**) C 1s spectra, and (**E**, **F**) Fe 2p spectra of fresh Fe-Mo_2C/ZSM-5 and Cat-1. The assignments of XPS Fe 2p were based on the data provided in the handbook and previous studies: Fe^0 $2p_{3/2}$ (~707 eV), Fe^{2+} or Fe^{3+} $2p_{1/2}$ (~725 eV) and $2p_{3/2}$ (~710 eV).

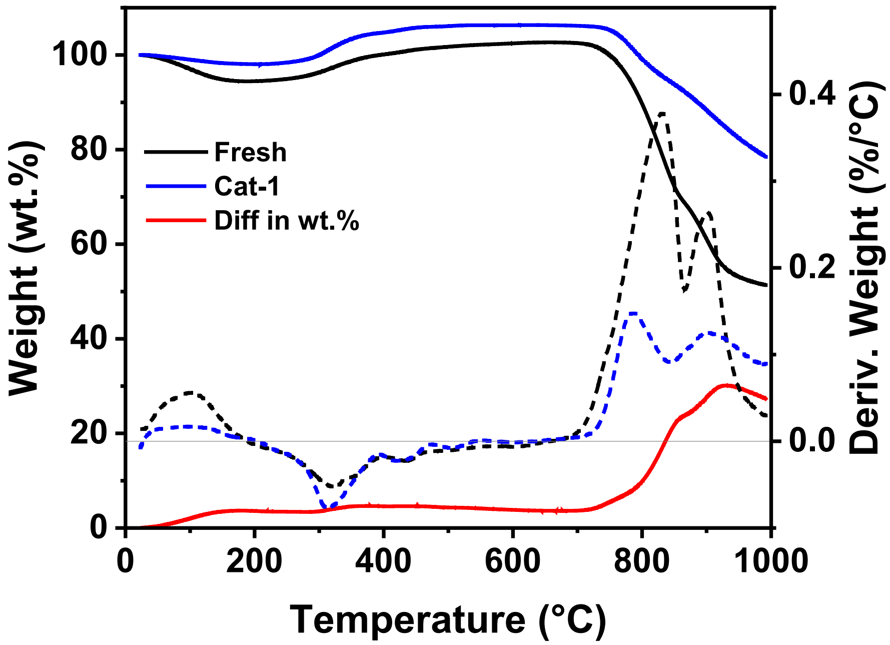

Fig. S19. Thermogravimetric analysis (TGA) curves of freshly made and re-used Fe-Mo_2C/ZSM-5 catalysts. The weight (wt.%) losses and their corresponding derivative over temperature of freshly made and re-used Fe-Mo_2C/ZSM-5 catalysts are shown.

Tables

Table S1. Contents of Mo and Fe in Fe-Mo_2C/ZSM-5 catalyst obtained from inductively coupled plasma optical-atomic emission spectroscopy (ICP-OES) analysis.

Content (wt.%)	Fe	Mo
Freshly prepared, passivated	1.30±0.54 [a]	41.9±2.8
Post-upgrading (Cat-1)	1.98±0.08	41.7±7.3
Theoretical [b]	1.26	43.1

[a] Error bar is calculated from results from three parallel measurements using identical samples.

[b] Theoretical values are calculated based on the feeding amount of metal precursors as listed in **Materials and Methods** Section, assuming a final product that consists of ZSM-5, Mo_2C, and Fe.

Table S2. Size estimations of nanocrystals based on Rietveld refinement [a] of XRD pattern for Fe-Mo_2C/ZSM-5.

	LVol-IB [b] (nm)	Error [c] (nm)
Mo_2C	3.04	0.04
ZSM-5	62	2

[a] The crystallographic information file (cif) used in the refinement can be found in Crystallography Open Database (COD). The COD ID is 5910009 for Mo_2C and 1505106 for ZSM-5.

[b] Volume-weighted mean crystallite size, as defined in TOPAS, a profile fitting software, was provided by Bruker Corporation.

[c] Error is calculated by the TOPAS software.

Table S3. CHN analysis, HHV, and the degree of deoxygenation for the raw and its upgraded oils under different conditions.

	Raw [b]	No Cat. [b]	Comm. [b]	Fe-Mo_2C/ZSM-5 [c]
C (wt.%)	80.35±0.10	84.75±1.32	77.61±1.18	85.66±0.15
H (wt.%)	12.26±0.03	12.98±0.25	11.98±0.17	14.08±0.09
O (wt.%)	6.74±0.08	1.70±1.53	9.79±1.48	-0.06±0.17
N (wt.%)	0.64± 0.06	0.57±0.08	0.62±0.15	0.32±0.03
H/C (mol/mol)	1.82±0.01	1.82±0.01	1.84±0.01	1.96±0.01
Deoxygenation (%)	-	74.81±22.64	-	100.93±2.5
HHV[a] (MJ/kg)	41.80±0.06	44.73±0.91	40.17±0.77	46.51±0.12

[a] The HHV is calculated based on Dulong's formula:

$$HHV\left(\frac{MJ}{kg}\right) = 0.3516 \times C + 1.16225 \times H - 0.1109 \times O + 0.0628 \times N$$

[b] Error bars were obtained based the calculation of standard deviation from six measurements after dropping the outlier value. A value is considered as an outlier, if it is 1.5 times more than that of interquartile range (IQR) below the first quartile or more than 1.5 IQR above the third quartile.

[c] Error bars for upgraded oils using Fe-Mo_2C/ZSM-5 were obtained based the calculation of standard deviation of four measurements. No outlier was found in these measurements.

Table S4. Elemental (CHN) analysis, H/C value and HHV of Jet A.

	Jet A
C (wt.%)	85.61±0.50 [a]
H (wt.%)	13.74±0.18
O (wt.%)	0.05±0.69
N (wt.%)	0.61±0.02
H/C (mol/mol)	1.91±0.01
HHV (MJ/kg)	46.10±0.46

[a] Error bars were obtained based on the standard deviation from of three measurements using the same sample.

Table S5. TGA results for the raw and its upgraded oils under different conditions *

T (°C)	Raw	No Cat.	Comm.	Fe-Mo$_2$C/ZSM-5
<100	4.58	13.4	12.8	14.2
100~150	7.09	19.6	18.1	17.5
150~200	14.6	29.0	28.7	32.0
200~250	25.3	19.6	21.4	30.6
250~300	41.0	9.70	10.4	5.34
300~350	6.53	4.99	5.40	0.14
>350 [a]	0.9	3.7	3.2	0.2

<table>
<tr><th>Oil type</th><th>Raw</th><th>No Cat.</th><th>Comm.</th><th>Fe-Mo$_2$C/ZSM-5</th></tr>
<tr><td>Gasoline</td><td>11.5</td><td>32.5</td><td>30.4</td><td>31.3</td></tr>
<tr><td>Kerosene</td><td>29.7</td><td>43.7</td><td>45.1</td><td>57.4</td></tr>
<tr><td>Diesel</td><td>57.8</td><td>19.6</td><td>20.7</td><td>11.1</td></tr>
<tr><td>Lubricating oils</td><td>0.26</td><td>1.69</td><td>1.69</td><td>0.01</td></tr>
<tr><td>Fuel oils</td><td>0.49</td><td>2.08</td><td>1.69</td><td rowspan="2">0.2</td></tr>
<tr><td>Residue [b]</td><td>0.2</td><td>0.4</td><td>0.4</td></tr>
</table>

* The weight distribution (wt.%) was categorized based on the temperature ranges at an interval of 50 °C and the types of oils.

[a, b] Values in these two rows were calculated by the differences in elemental analysis. All other values are integrals of percentage weight loss over the corresponding temperature range using Universal Analysis 2000 software, provided by TA Instruments, Waters LLC.

Table S6. TGA results of Jet A.

T (°C)	Jet A
<100	16.6
100~150	59.4
150~200	23.9
200~250	0.019
250~300	0.017
300~350	0.014
>350 [a]	0.05

Oil type	Jet A
Gasoline	74.5
Kerosene	25.4
Diesel	0.03
Lubricating oils	0.01
Fuel oils	0.06
Residue [a]	0

[a] Values in these two rows were calculated by the differences in elemental analysis. All other values are integrals of percentage weight loss over temperature using Universal Analysis 2000 software, provided by TA Instruments, Waters LLC.

Table S7. Number-weighed molecular weights distribution, number-averaged molecular weight (M_n), mass-averaged molecular weight (M_m), and polydispersity index (I) calculated from MALDI-TOF-MS results for the raw and its upgraded oils under different conditions.

Weight (Da)	Raw	No Cat.	Comm.	Fe-Mo_2C/ZSM-5	Jet A [b]
<100	-0.5 [a]	0.0	0.0	1.3	0.1
100~200	4.9	0.4	2.8	14.8	33.8
200~300	21.0	7.6	20.2	44.6	28.3
300~400	16.8	11.9	22.3	24.4	16.0
400~500	13.9	16.1	20.1	7.8	5.6
500~600	11.6	16.1	13.1	3.1	7.8
600~700	9.8	13.8	8.1	1.6	3.2
700~800	8.0	11.5	5.4	0.9	2.4
800~900	6.2	9.1	3.5	0.6	1.3
900~1000	4.5	7.1	2.4	0.4	0.9
>1000	3.9	6.4	2.2	0.4	0.7
M_n	404	522	389	254	242
M_m	500	602	463	303	313
I	1.24	1.15	1.19	1.19	1.29

[a] Negative value is due to the baseline correction, resulted from the signals subtracting the average noise level determined by the observed plateau in the mass spectra (**fig. S12**), normally >1000 Da.

[b] Data of Jet A is included for comparison.

Table S8. Measured density (ρ) for the raw and its upgraded oils under different conditions.

	Raw	No Cat.	Comm.	Fe-Mo_2C /ZSM-5	Jet A [b]
ρ (g/mL)	0.853±0.009 [a]	0.823±0.004	0.812±0.007	0.783±0.009	0.796±0.002

[a] Error bar is the standard deviation of three parallel measurements of the same samples.

[b] Data of Jet A is included for comparison.

Table S9. CHN analysis, HHV, and the degree of deoxygenation for upgraded oils using freshly made and re-used Fe-Mo_2C/ZSM-5 catalysts.

	Raw [a]	Oil-1	Oil-2 [b]	Oil-3 [b]
C (wt.%)	80.35±0.10	85.66±0.15	86.40±0.30	86.68±0.73
H (wt.%)	12.26±0.03	14.08±0.09	13.74±0.13	13.85±0.09
O (wt.%)	6.74±0.08	-0.06±0.17 [c]	-0.80±0.36	-1.29±0.71
N (wt.%)	0.64± 0.06	0.32±0.03	0.66±0.03	0.77±0.09
H/C (mol/mol)	1.82±0.01	1.96±0.01	1.90±0.02	1.90±0.01
Deoxygenation (%)	-	100.9±2.5	111.9±5.3	119.2±10.5
HHV (MJ/kg)	41.80±0.06	46.51±0.12	46.48±0.23	46.76±0.42 [d]

[a] Data are included as references.

[b] Errors for Oil-2 and Oil-3 are standard deviations of three measurements using the same samples.

[c] Negative values were obtained because the oxygen content was calculated by the differences.

[d] When Dulong's formula is used, a negative oxygen content may result in inaccurate value of HHV, though the difference is insignificant and should not affect the general conclusion on the properties of the oil products. For example, the HHV of Oil-3 was calculated to be 46.62±0.34 MJ/kg, if 0%, instead of -1.29 wt.%, of oxygen was used.

Table S10. Measured density (ρ) for upgraded oils using fresh or reused Fe-Mo_2C/ZSM-5.

	Raw [a]	Oil-1	Oil-2	Oil-3
ρ (g/mL)	0.853±0.009	0.783±0.009	0.779±0.009	0.783±0.009

[a] Data of raw oil is included as reference. Error was obtained from the standard deviation of three measurements using the same samples.

Table S11. TGA results of upgraded oils using freshly made and re-used Fe-Mo_2C/ZSM-5.

T (°C)	Raw	Oil-1	Oil-2	Oil-3
<100	4.59	14.2	13.7	12.0
100~150	7.09	17.5	18.9	14.6
150~200	14.6	32.0	35.5	21.8
200~250	25.3	30.6	25.8	34.0
250~300	41.0	5.34	5.23	16.1
300~350	6.53	0.14	0.64	1.28
>350 [b]	0.9	0.2	0.2	0.2

<table>
<tr><th>Oil type</th><th>Raw</th><th>Oil-1</th><th>Oil-2</th><th>Oil-3</th></tr>
<tr><td>Gasoline</td><td>11.5</td><td>31.3</td><td>32.1</td><td>26.4</td></tr>
<tr><td>Kerosene</td><td>29.7</td><td>57.4</td><td>55.7</td><td>44.4</td></tr>
<tr><td>Diesel</td><td>57.8</td><td>11.1</td><td>12.0</td><td>29.0</td></tr>
<tr><td>Lubricating oils</td><td>0.26</td><td>0.01</td><td>0.03</td><td>0.03</td></tr>
<tr><td>Fuel oils</td><td>0.49</td><td rowspan="2">0.2</td><td>0.08</td><td>0.01</td></tr>
<tr><td>Residue [b]</td><td>0.2</td><td>0.1</td><td>0.2</td></tr>
</table>

[a] Data of raw oil are included as references.

[b] Values in these two rows are calculated based on the differences. All other values are integrals of percentage weight loss over the corresponding temperature range using Universal Analysis 2000 software, provided by TA Instruments, Waters LLC.

Table S12. Size estimations of Mo_2C nanocrystals based on Rietveld refinement of XRD pattern for used Fe-Mo_2C/ZSM-5 catalysts.

	LVol-IB (nm)	Error (nm) [a]
Cat-1	2.09	0.19
Cat-2	1.29	0.23
Cat-3	1.35	0.09

[a] Errors are calculated using the TOPAS software.

Table S13. Results of XPS analyses using software CasaXPS.

Fresh Fe-Mo_2C/ZSM-5					
Name	Position (eV)	ΔBE (eV)	%Area	Area ratio	FWHM
C=O	288.79	/	3.70	/	1.09
C-O	285.18	/	41.48	/	2.80
ADC [b]	284.80	/	52.28	/	1.15
C-Mo	283.56	/	2.55	/	0.68
Mo(VI) $3d_{3/2}$	235.62	2.76	16.20	0.727	2.53
Mo(VI) $3d_{5/2}$	232.86	/	22.28	/	2.05
Mo(IV) $3d_{3/2}$	232.07	2.57	13.21	0.691	1.85
Mo(IV) $3d_{5/2}$	229.50	/	19.12	/	1.73
Mo(II) $3d_{3/2}$	231.82	3.18	10.60	0.570	1.24
Mo(II) $3d_{5/2}$	228.64	/	18.59	/	1.05
**Cat-1**					
Name	Position (eV)	ΔBE (eV)	%Area	Area ratio	FWHM
C=O	288.74	/	4.25	/	1.41
C-O	285.25	/	50.56	/	3.10
ADC [b]	284.80	/	41.15	/	1.27
C-Mo	283.41	/	4.03	/	0.65
Mo(VI) $3d_{3/2}$	235.85	2.75	13.84	0.682	1.71
Mo(VI) $3d_{5/2}$	233.11	/	20.28	/	1.83
Mo(IV) $3d_{3/2}$	232.37	2.95	13.62	0.720	1.48
Mo(IV) $3d_{5/2}$	229.42	/	18.91	/	2.00
Mo(II) $3d_{3/2}$	231.66	3.08	14.50	0.769	1.33
Mo(II) $3d_{5/2}$	228.58	/	18.85	/	1.00

[a] Values of XPS position, %Area, and FWHM were obtained using the CasaXPS software. Values in columns of ΔBE and Area ratio were calculated based on the output.

[b] ADC: adventitious carbon.
